# Morning boost on individuals’ psychophysiological wellbeing indicators with supportive, dynamic lighting in windowless open-plan workplace in Malaysia

**DOI:** 10.1371/journal.pone.0207488

**Published:** 2018-11-29

**Authors:** RatnaKala Sithravel, Rahinah Ibrahim, Munn Sann Lye, Enoch Kumar Perimal, Normala Ibrahim, Nur Dalilah Dahlan

**Affiliations:** 1 Department of Architecture, Faculty of Design and Architecture, Universiti Putra Malaysia, Serdang, Selangor, Malaysia; 2 Department of Community Health, Faculty of Medicine and Health Sciences, Universiti Putra Malaysia, Serdang, Selangor, Malaysia; 3 Department of Biomedical Sciences, Faculty of Medicine and Health Sciences, Universiti Putra Malaysia, Serdang, Selangor, Malaysia; 4 Department of Psychiatry, Faculty of Medicine and Health Sciences, Universiti Putra Malaysia, Serdang, Selangor, Malaysia; Università Cattolica del Sacro Cuore, ITALY

## Abstract

Workplace architectural lighting conditions that are biologically dim during the day are causing healthy individuals to experience light-induced health and performance-related problems. Dynamic lighting was reported beneficial in supporting individuals’ psychological behavior and physiological responses during work period in Europe. It has yet to be investigated in workplaces with minimal/no natural daylight contribution in tropical Malaysia. Hence, an exploratory experimental study was initiated in an experimental windowless open-plan workplace in Universiti Putra Malaysia, Serdang. The aim was to identify dynamic lighting configurations that were more supportive of a morning boosting effect than the control constant lighting, to support dayshift individuals’ psychophysiological wellbeing indicators during the peak morning work period. The immediate impact of a 2-hour morning exposure to overhead white LED (6500 K) with different horizontal illuminance levels and oscillations (lighting patterns) were investigated on physiological indicator limited to urinary 6-sulfatoxymelatonin, and psychological indicators for alertness, mood, visual comfort, cognitive and visual task performance. Not all of the investigated dynamic lighting configurations were supportive of a morning boost. Only configurations 500_increased to_750 and 500_increased to_1000 lx therapeutically supported most of the indicators. Both these configurations suppressed urinary 6-sulfatoxymelatonin, and improved alertness, cognitive performance, positive affect, and visual comfort better than ‘visit 1: 500_constant_500’ lx (control). The increasing oscillation was observed more beneficial for the morning boost in tropical Malaysia, which is in reverse to that specified in the human rhythmic dynamic lighting protocol developed by researchers from the Netherlands for application during winter. The findings from this study present the feasibility of dynamic architectural lighting acting as an environmental therapeutic solution in supporting the individuals’ psychophysiological wellbeing indicators in windowless open-plan workplace in tropical Malaysia. Further investigations on the two prospective configurations are recommended to determine the better supportive one for the morning boosting effect in Malaysia.

## Introduction

Architectural (artificial) lighting is a necessary utility in workplaces. According to research agencies in World Health Organization, architectural lighting exposures are relatively dim during the day (mainly < 500 lx in workplace [1]) and too bright at night when compared to the natural daylight and moonlight levels. These 2 exact opposite environmental lighting scenarios are contributing towards light-induced circadian disruption (circadian desynchronization from an entrained condition due to the inappropriate architectural lighting exposures throughout the day) [2–4]. Living in biological darkness during the day has also been reported to cause light-induced health and performance-related problems [5].

Hence, the wellbeing of Malaysian dayshift individuals who work in windowless open-plan workplace (WOPW) is a concern, as they could suffer from light-induced circadian disruption. The WOPW setting is mostly observed in the intermediate shop-lots and office towers, where the deep-plan layout creates a mid-zone windowless workplace with minimal/no natural daylight contribution. Individuals working in WOPW are not only exposed to prolonged biologically dim and constant architectural lighting conditions, but also deprived of the natural daylight essential for their wellbeing. Therefore, there is a need to identify architectural lighting conditions that support dayshift individuals’ psychophysiological wellbeing indicators (IPWI) in WOPW in Malaysia.

Andersen et al. [6] reported the characteristics of light, and its timing of exposure determines the magnitude and direction of the circadian resetting effect, besides influencing acute alerting effects. Exposure to bright blue-enriched light from early to mid-morning (6am to 11am) has been reported to act as a powerful stimulus to counter light-induced circadian disruption and stabilize the circadian phase [3,6,7]. Moreover, compared to dim light (200 lx, 4000 K), exposure to bright light (1000 lx, 4000 K) resulted in improved alertness and vitality in the morning, but not necessarily in the afternoon [8,9]. These studies provided evidence that planning the workplace light exposure is critical to support the different daytime requirements of IPWI according to the timing, purpose, and type of activity performed [6].

As an innovative approach to minimize light-induced circadian disruption during the day, this study referred to the human rhythmic dynamic lighting protocol developed by van den Beld [10], and van Bommel [11,12] from the Netherlands. The luminous conditions in the protocol were designed with the intention to support and even enhance dayshift individuals’ natural rhythm of alertness [13] and positively impact wellbeing and productivity during winter [11]. It recommended timely exposure to changing horizontal illuminance (E_H_) and color temperature (CCT) for the activation-relaxation of the IPWI during the workday.

**Fig 1:**
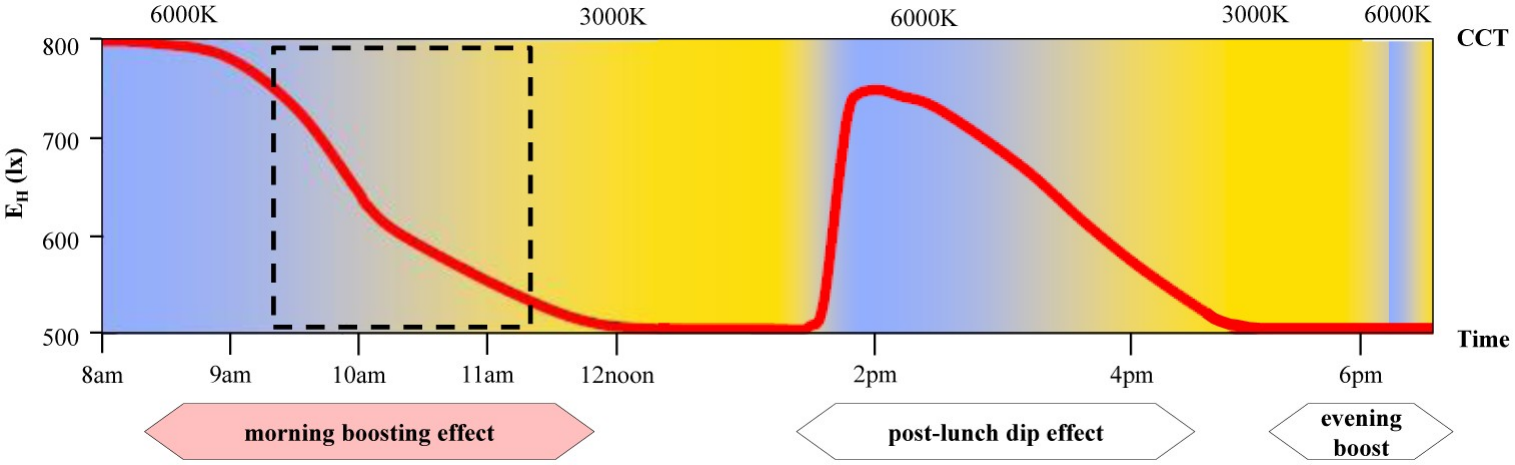
The human rhythmic dynamic lighting protocol. Red solid line represents gradual changes of illuminance levels, Blue shaded area represents CCT 6000 K, Yellow shaded area 3000 K, Black dotted line focuses on the recommended luminous conditions for boosting effect during the peak morning work period. (With permission from [12]).

The protocol by van Bommel [11,12] (Fig 1) specified workplace ambient lighting to start with 800 lx (6000 K) around 8am–9am. The bright luminous condition was provided to regulate the 24-hour circadian rhythm as individuals arrived at work under dark conditions during winter. The luminous conditions then gradually decline to 500 lx (3000 K) during lunch-time to create an emotionally relaxing atmosphere in the afternoon. Around 2pm a sharp increase in E_H_ and CCT is introduced for re-activation and compensate the post-lunch dip effect. It then gradually drops to 500 lx (3000 K) at 6pm for relaxing effect. Before clocking out, either a brief increase in E_H_ or exposure to 6000 K is given as a booster to freshen-up and cope with evening fatigue [10–12]. As highlighted by van Bommel [12], an initial small-scale practical testing under the human rhythmic dynamic lighting conditions indicated beneficial effects, such as improved alertness and its acceptance. With that, van Bommel [12] recommended further large-scale empirical investigations to document the effects of the human rhythmic dynamic lighting installations on wellbeing, preference, and productivity, to improve and refine the lighting configurations.

In the first large-scale field test that investigated the effects of the human rhythmic dynamic and constant architectural lighting conditions on office workers, de Kort & Smolders [13] found no significant differences in the need for recovery, vitality, alertness, headache and eyestrain, mental health, sleep quality, and subjective performance between both the lighting conditions, except for employees feeling more satisfied under the dynamic lighting condition. Their study was not able to separate the contribution of the substantial natural daylight from the effects of human rhythmic dynamic and constant architectural lighting conditions. Even though their study was performed during the darker months of the year, the substantial uncontrolled natural daylight contribution from the large windows could have confounded the relationship between the 2 lighting conditions and the study outcomes. As such, their mainly null intermediate results of the human rhythmic dynamic lighting conditions on office workers in a setting with extensive daylight have to be considered with caution. Nevertheless, in settings with minimal/no natural daylight contribution, other studies from the European context [14–16] found dynamic lighting during work period was beneficial in supporting individuals’ psychological behavior and physiological responses when compared to constant lighting. Dynamic lighting was also suggested most effective for supporting various IPWI in workplace with less natural daylight contribution [7,13].

The concern is how to configure supportive, dynamic lighting for Malaysia. The abovementioned dynamic lighting studies were mainly from the European context. They had developed and tested their dynamic configurations accommodating for seasonal climates. Their configurations may not necessarily be applicable for the tropics due to genetic, geographical location, climate, and cultural differences. To the best of the authors’ knowledge, no local dynamic lighting configurations have been defined yet for the tropics. Hence, an exploratory experimental study was initiated in Universiti Putra Malaysia (UPM), Serdang, in an attempt to provide the initial empirical evidence to develop the human rhythmic dynamic lighting configurations for supporting dayshift IPWI in WOPW setting in tropical Malaysia. Since there were no local studies to refer to, this study investigated a few dynamic lighting configurations comprising of different E_H_ levels (illuminance on the desk plane corresponding with office task level) and oscillations (lighting patterns) that are likely to impact the measured IPWI during the peak morning work period.

The aim was to identify dynamic lighting configurations that could be more supportive of the morning boosting effect than the control constant lighting, so as to support dayshift IPWI during the peak morning work period in WOPW in Malaysia. A supportive configuration has the potentials to support most of the measured IPWI from the 3 separate yet interrelated routes [17] that influence individuals’ psychophysiological wellbeing, and be in the direction needed for a morning boost during the peak morning work period. The 3 separate yet interrelated routes [17] include the circadian system route and mood route related to the complex non-image-forming system, and visual system route related to the image-processing system [11,18].

**Fig 2:**
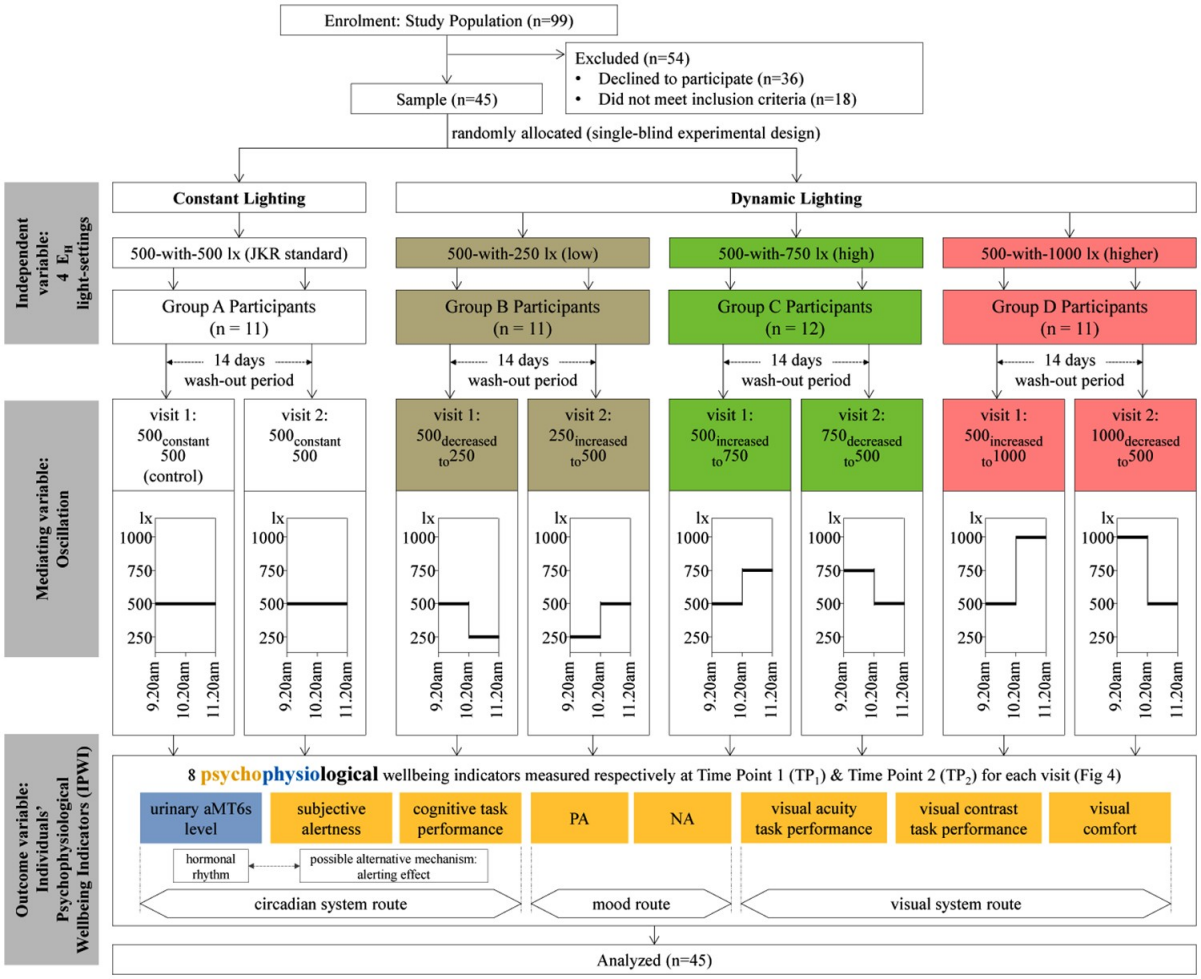
Schematic diagram describing the variables and experimental study design.

Boyce [17] highlighted the circadian system route has many mechanisms which remain as possibilities, and have yet to be fully comprehended to be distinctly defined. The circadian system route regulates the 24-hour rhythm of the physiological responses like melatonin rhythm, and psychological behavior like alertness, sleep-wake cycle [17]. It is possibly linked with an alternative mechanism (alerting effects) that influences daytime alertness and cognitive performance [9,19]. Next, the mood route influences the emotional responses, while the visual system route influences the seeing process for visual task performance and preference [17]. Fig 2 presents the measured IPWI in this study. They include urinary 6-sulfatoxymelatonin (aMT6s) the major metabolite of melatonin, subjective alertness, and cognitive task performance to relate to the circadian system route; positive affect and negative affect to relate to the mood route; and visual acuity-contrast task performance and visual comfort to relate to the visual system route.

## Materials and methods

### Ethics approval

Ethics Committee for Research Involving Human Subjects, Universiti Putra Malaysia, approved this study (Approval Reference: FRBS(EXP15)P185). Written informed consent was obtained from all participants.

### Study design

**Fig 3:**
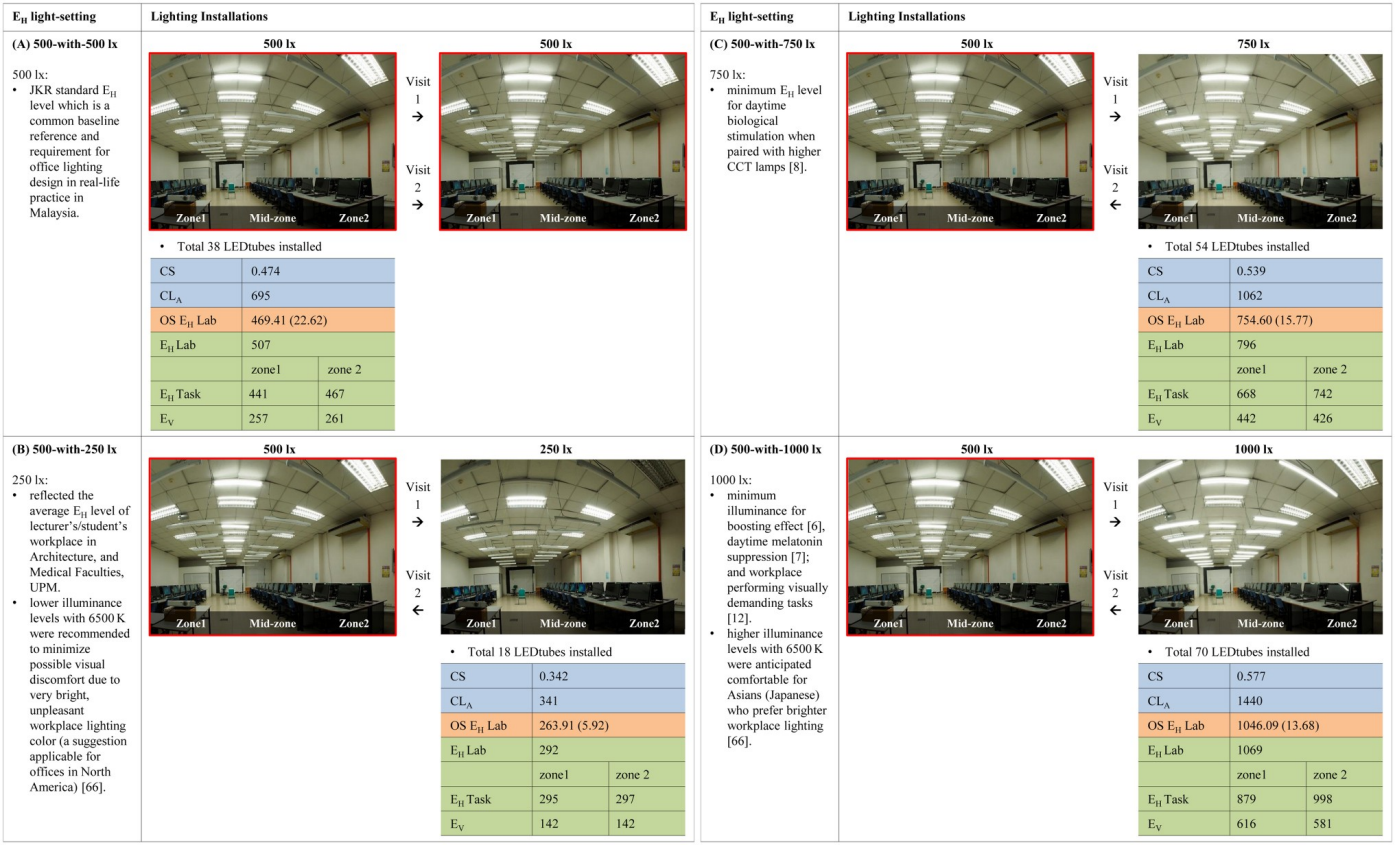
Lighting installation and characteristics of each E_H_ light-setting. (A) 500-with-500 lx. (B) 500-with-250 lx. (C) 500-with-750 lx. (D) 500-with-1000 lx. Public Works Department Malaysia (JKR), Red border indicates similar OS E_H_ Lab levels. Blue shaded values are the Circadian Stimulus Estimation, estimated with CS metric calculator: Circadian Stimulus value (CS), Circadian Light (CL_A_) circadian lx. Light orange shaded values are the On-Site Measurement performed during each experimental session with the 13 monitors switched on, measured with luxmeter: Mean (SD) horizontal illuminance at 0.755 m from FFL in the mid-zone area (OS E_H_ Lab) lx. Green shaded values are the DIALux Simulated Model Estimation, estimated with DIALux: Average horizontal illuminance at 0.755 m from FFL in the mid-zone area (E_H_ Lab) lx, Average horizontal illuminance at 0.755 m from FFL at Zone1 and Zone2 (E_H_ Task) lx, Average vertical illuminance at 1.105 m from FFL at Zone1 and Zone2 (E_V_) lx.

An experimental study design (Fig 2) was employed to investigate the immediate impact of a 2-hour morning exposure to overhead white LED (6500 K) ambient lighting on the measured IPWI. There were 4 independent E_H_ light-settings: 500-with-500 lx as constant lighting (Public Works Department Malaysia (JKR) standard), and 500-with-250, 500-with-750, 500-with-1000 lx as dynamic lighting representing ‘low’, ‘high’ and ‘higher’ interventions. The justifications for the predetermined 500, 250, 750, and 1000 lx E_H_ levels are presented in Fig 3. Each of the 4 E_H_ light-setting had its group of randomly allocated participants. The participants within each E_H_ light-setting were then exposed to different oscillations scheduled on 2 separate days.

**Fig 4:**
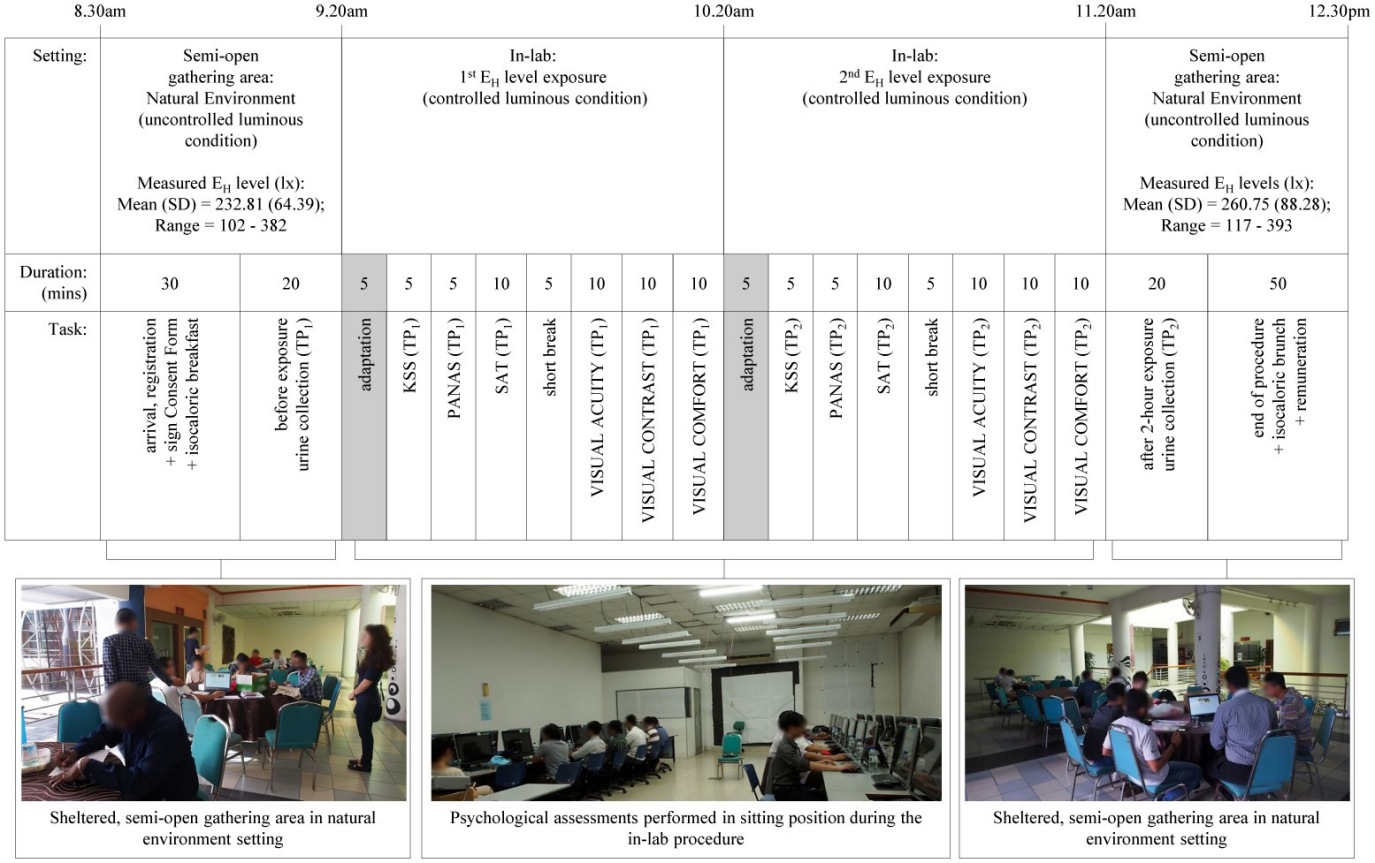
Details of the experimental procedure for each session.

Oscillation refers to the lighting pattern (direction of change between the 2 E_H_ levels in each light-setting). The constant lighting had 500 lx throughout the 2-hours on visits 1 and 2. The 500_constant_500 on visit 1 served as control, while visit 2 as a follow-up comparison to control. As for the dynamic interventions, each light-setting had either a decreasing or increasing E_H_ levels on visits 1 and 2, counterbalanced within the same group of participants to understand the immediate effects due to lighting patterns. During each visit, physiological indicator limited to urinary aMT6s was measured before (Time Point 1, TP_1_) and after (Time Point 2, TP_2_) the 2-hour in-lab procedure. Psychological indicators for subjective alertness, mood, visual comfort, cognitive and visual acuity-contrast task performance were respectively measured in the 1^st^ (TP_1_) and 2^nd^ (TP_2_) E_H_ level exposure during the in-lab procedure (Fig 4). Each indicator’s immediate change (from TP_1_ to TP_2_) was compared in all the light-settings, as well as, the immediate impact of each light-setting on the indicator in comparison to ‘visit 1: 500_constant_500’ lx as the control.

Comparing the 4 E_H_ light-settings, this study hypothesized the ‘higher’ (500-with-1000) and ‘high’ (500-with-750) dynamic lighting would have greater magnitude of impact than the constant lighting (500-with-500), while the ‘low’ (500-with-250) would have less. Studies that investigated the effects of bright versus dim light on the IPWI in settings without natural daylight contribution/windowless reported higher illuminance levels (brighter lighting) led towards bigger magnitude of melatonin suppression [20,21], and improvement in alertness [8,9], mood [22] and cognitive task performance [9]. Therefore for each indicator, the 1000_decreased to_500 and 500_increased to_1000 (both of 500-with-1000), followed by 750_decreased to_500 and 500_increased to_750 (both of 500-with-750) would contribute towards a more supportive impact than the control, while ‘visit 2: 500_constant_500’ (of 500-with-500), and 500_decreased to_250 and 250_increased to_500 (both of 500-with-250) lx would be less supportive. Since each dynamic intervention had 2 oscillations, the decreasing oscillation was hypothesized to have bigger magnitude of impact than its increasing counterpart, relating to the protocol’s decreasing E_H_ levels for a morning boosting effect [10–12]. ‘Visit 2: 500_constant_500’ lx would be less supportive than control due to the unvarying light stimuli over time.

A minimum sample size of 10 participants was estimated for each of the 4 E_H_ light-setting based on the 2-groups comparison calculation and Power Analysis Chart (Lemeshow et al., 1990; Lipsey, 1990 in [23]). The reported mean (SD) of the excreted urinary melatonin levels of the morning (before 9am) and afternoon (around 4pm) samples that showed a stable and highly significant diurnal variation in Küller & Wetterberg [24] were incorporated into the 2-groups comparison calculation formula. The calculated standardized effect size was 1.93, and the estimated sample size of 10 participants for each E_H_ light-setting was determined from the power analysis chart reflecting the closest effect size of 2.00, with power = 90%, α = 0.05. An additional 10% anticipated for dropout rate hence required 45 participants for this exploratory experimental study.

Malaysian, male postgraduates from the engineering and life sciences faculties/institution of UPM (S1 Table) were invited to participate through email and manually distributed invitation forms. Participants were restricted to males (controlled for gender by restriction) to avoid confounding menstrual cyclicity with circadian cyclicity [25]. A total of 667 invitations were sent, of which 99 postgraduates responded. They were screened via a screening questionnaire to determine their eligibility based on age, chronotype, lifestyle habits, and general health state. Only non-smoking, healthy volunteers (not physically or mentally impaired) aged between 20 to 35 years (controlling them for similar melatonin excretion levels by age [26]); and scored 31 to 69 as ascertained by the adapted Horne–Östberg Morningness-Eveningness Questionnaire (MEQ) [27,28] were selected. They also had to be without current medication, and neither worked night shifts nor traveled > 3 time zones 2-months before the scheduled experimental sessions.

Forty-five postgraduates who fulfilled the above criteria attended a briefing session. They were randomly allocated to the 4 E_H_ light-settings following a simple randomization procedure. Before the briefing session, 48 identification numbers (ID = 001 to 048) were generated and allotted equally to the 4 E_H_ light-settings via a draw performed by the researcher. A reference list was prepared containing information on the scheduled experimental sessions (2 separate dates) with 12 IDs randomly assigned to each of the 4 E_H_ light-setting (S2 Table). During the briefing session, each participant drew his ID from an opaque container containing the 48 IDs. With the participant’s ID, the researcher identified his assigned setting and experimental sessions from the reference list prepared beforehand. This randomization procedure resulted in 12 participants in 500-with-750, and 11 participants each in 500-with-500, 500-with-250 and 500-with-1000 lx. It also ensured each participant had an equal chance of being assigned to any one of the 4 E_H_ light-settings, thus generating comparable groups between the settings and minimizing selection bias [23,29].

Participants were briefed that as a group they will be exposed to different lighting conditions during each visit. However, during the briefing session, each participant was only informed of his scheduled dates for visits 1 and 2 to maintain the single-blind process. Information on allocation into control-intervention groups, the assigned E_H_ light-setting, and counterbalanced oscillation were withheld. It was to safeguard against performance bias where results on the subjective assessments could either be overestimated or underestimated due to prior knowledge of the exact lighting conditions [13,30].

### WOPW experimental setting

The 2-hour in-lab procedure was conducted in a computer laboratory located on the 1^st^ floor of Faculty of Design and Architecture, UPM. This laboratory mimicked a close to real-life WOPW setting without partitions separating the workstations. It had no natural daylight contribution and no additional localized task lighting during the procedure. Table 1 describes the laboratory’s detail setup.

**Table 1:** Laboratory’s condition throughout the study duration.

**Area**	14 m (L) x 9 m (W) = 126 m^2^			
**Ceiling height**	3.65 m			
**Temperature**	Mean (SD) = 24.17 (0.57) °C; regulated via split air-conditioning units			
**Relative Humidity**	Mean (SD) = 51.36 (4.48) %			
**Laboratory’s Finishes and Main Furnishing** (Measured with a reflectance sample card (SLL LG11 Color Chart, CIBSE, London))	**Surface**	**Material**	**Color**	**Reflectance (%)**
	ceiling	perforated acoustic gypsum ceiling boards	neutral white	80
	wall	painted brick wall, plasterboard	neutral white	81
	glazing	screened with paper sheets (white mahjong over black sugar paper)	whitish grey	79
	floor	matt finish porcelain tiles	light cream	40
	desk	matte finish laminate on compressed wood	light grey	36
	chair	matte finish plastic seater	dark blue	17

There were 13 workstations arranged side by side in 2 opposite rows (facing the wall). All workstations had an identical 23-inch LED-backlit monitor set at its maximum brightness. The mean (SD) luminance of its blank white screen display was 133.82 (15.67) lx with color temperature 6415.05 (106.67) K measured using a portable digital spectrometer (UPRtek MK350N LED Meter, Taiwan). These readings were measured at 1.105 m (average participant’s eye level in sitting position) from finished floor level (FFL), with a viewing distance of 0.55 m from the monitor, when the laboratory’s E_H_ level was 458.85 (16.63) lx and color temperature 6425.85 (16.61) K.

The monitors’ position was fixed to maintain constant workplace setting throughout the procedure and was tilted 10° from vertical to minimize the visibility of the overhead luminance reflections on the screen. The procedure used a free-seating concept to reflect an open-plan occupancy pattern. The participants were allowed to sit at any one of the 13 workstations for each visit. They were not allowed to switch workstations during the procedure. All participants sat at the same workstation zones on both visits, except for 2 participants who preferred a switch during visit 2.

#### Overhead white LED (6500 K) ambient lighting

The overhead light fittings were fitted with Philips MASTER LEDtube EM 1200 mm 18W865 T8 I lamps (Rated Luminous Flux: 2100 Lm, Material No.: 929001178708, Philips Malaysia Sdn. Bhd.). S1 Appendix presents the specifications of this white LED lamp (CCT of 6500 K, color rendering index of 83, and its spectral power distribution) which fulfilled this study’s requirement. Using white LED was timely and reasonable because the effects of dynamic lighting with such lamps on IPWI needed to be investigated. Rea [31] and Hawes et al. [32] foresee LED lamps will be the future of general workplace lighting, replacing the commonly used incandescent and T8 fluorescent lamps. Moreover, the Circadian Stimulus (CS) metric calculator estimated all the 4 E_H_ levels (500, 250, 750, and 1000 lx) with the abovementioned lamp achieved CS value > 0.3. As recommended by Rea & Figueiro [33], these values had circadian effective light characteristics that were appropriate for a morning period.

With that, a new, temporary overhead lighting circuit was designed to accommodate the 4 circadian effective E_H_ levels. The best fitting layout was computationally determined using DIALux Evo (DIAL, GmbH, Germany). The existing ceiling mounted parabolic-louvers fitting at 3.65 m from FFL and additional suspended bare channel holders at 2.70 m from FFL provided the laboratory’s general and localized direct lighting. The underside of the suspended fittings was > 2.30 m from FFL which provided sufficient head height to achieve good uniformity and minimize the glare at task level [34].

The researcher controlled the laboratory’s ambient lighting by switching on/off specific light switch for each E_H_ level. This action caused an immediate transition in the luminous condition. The rectangular change approach was implemented because a step function was reported more efficient for boosting effect compared to a gradual change in the illuminance levels [10]. Exposure to dynamic lighting with abruptly increasing illuminance resulted in lower urinary aMT6s level at 2am and 7am than the gradual change scenario [35]. Moreover, the rectangular change was posited reasonable for visual appraisal because the illuminance levels used in this study was less contrasting when compared to prior studies [14–16] that had gradual change for their extreme contrasting levels (difference of ± 1000 lx from a standard level).

Vertical illuminance (E_V_) levels were computationally estimated with DIALux (Fig 3). They were not measured [13,16,24] because illuminance at eye level in an open-plan workplace setting could vary depending on the individual’s head-cum-eye motion, viewing direction, usage of glasses, distance from the light source and workstation location [13,36,37]. Moreover, the approach of this exploratory experimental study was to investigate the immediate impact of the predetermined light-settings that have been designed based on the E_H_ levels like any practical architectural exercise (the process from schematic design to pre-occupancy installation stages). The E_H_ measurement is referred because it is still a widely used approach in real-life practice in the building industry [17,38,39]. Following this E_H_ approach, the CS metric calculator by Rea & Figueiro [33] was referred to ensure the overhead white LED ambient lighting complied with the CS value requirements, and did not pose any risk of designing biologically dim lighting for a morning period.

During each experimental session, the E_H_ levels were measured every 5 minutes in the middle of the laboratory using a portable digital luxmeter (Pro’sKit Digital Lightmeter, MT-4007, Taiwan). The E_H_ levels were similar between the mid-zone area and the workstations at Zone1 and Zone2. The false color distribution from the DIALux simulated model (S1 Fig) presents the symmetrical light distribution at task level. The mean (SD) of the measured E_H_ levels (OS E_H_ Lab) was 263.91 (5.92), 469.41 (22.62), 754.60 (15.77) and 1046.09 (13.68) lx for the respective 250, 500, 750 and 1000 lx. The ‘OS E_H_ Lab’ levels were similar for all 500 lx, and on both the visits for 250, 750 and 1000 lx, indicating a symmetrical change between the increasing and decreasing oscillations. Fig 3A–3D present the lighting installations and characteristics for each E_H_ light-setting.

### Procedure

The experimental sessions were held in February 2016. Fourteen days wash-out period was observed between visit 1 and 2 to minimize the carryover effects, participants’ awareness on the tested lighting scenario, and memory recall bias between assessments which may influence the results [23,29]. Fig 4 describes the executed experimental procedure for each session. The predetermined lighting conditions for each experimental session were only known to the researcher (S2 Table), and the 1^st^ E_H_ level was switched on (at 8.30am for each experimental session) before any participants entered the laboratory.

Before the experimental sessions, participants had been instructed (during the briefing session) to comply with a few pre-experiment instructions accommodative to real-life compliance. They were aware of and agreed to comply by signing the respondent’s information sheet agreement. The instructions included:

1. To maintain regular 7 to 8 hours’ sleep-wake schedule and avoid staying up late (e.g., beyond an estimated ± 30 minutes from their habitual sleep time) 5 days before each session. The bedtimes and wake times were left according to the discretion of each participant so that it was natural and matched their habitual sleep-wake schedule on workdays [8,40].
2. To avoid consumption of alcohol, coffee, tea, banana, cherries, and oatmeal with dairy products, 24-hours before each session.
3. To shade their eyes with sunglasses (or brimmed hat) at all times during their outdoor travels/movements on each session [41,42].

These were precautions to minimize any abnormal sleep-wake behavior, sleep deprivation, food substances and exposure to natural daylight influencing the endogenous melatonin rhythm and its daytime levels.

On each experimental session, participants gathered in a sheltered, semi-open gathering area (close to the laboratory and washroom) which had a natural environment setting (Fig 4). During registration, each participant signed his Consent Form and completed a short questionnaire on his ‘lifestyle habits’ before the experimental session. Participant’s adherence to the pre-experiment instructions during the past 24-hours was verified based on his self-declared answers in the questionnaire.

Twenty minutes before and soon after the 2-hour in-lab procedure, the participants were given their respective 50 mL sterile urine bottles to fill with 15 to 30 mL mid-stream urine. The collection time of each urine specimen was recorded. These samples were then immediately transported (upon each collection period) to a laboratory in Medical Faculty, UPM for aliquot and storage.

During the in-lab procedure from 9.20am to 11.20am (to coincide with the peak morning work period), participants completed all the computer-based psychological assessments in sitting position (Fig 4). Participants were not allowed to visit the washroom during the in-lab procedure. They were advised to drink mineral water (based on self-discretion) to produce enough urine at the end of the procedure if required. All participants received their remuneration upon signing out by 12.30pm.

### Measured IPWI

#### Urinary aMT6s (physiological indicator for circadian system route)

‘Single isolated urine sample’ collection method [43] was used to measure the urinary aMT6s concentration of a time point, a non-invasive procedure performed in a setting mimicking real-life situations and under natural environmental conditions which had similar E_H_ levels at TP_1_ and TP_2_ in the sheltered, semi-open gathering area (Fig 4). The before exposure samples were collected at mean (SD) 9.13am (0.11), while post-exposure samples at 11.24am (0.07). The urine samples were assayed for aMT6s concentration using a commercially available, highly sensitive, Melatonin Sulfate Urine ELISA kit (RE54031; IBL International, Hamburg, Germany), performed by a private lab-specialist (Prima Nexus Sdn. Bhd.) in UPM. The procedure follows the basic principle of competitive ELISA. The absorbance of each sample was read using the 450 nm wavelength in a microplate reader (FLUOstar Omega, BMG LABTECH GmbH, Germany). MyAssays online data analysis tool (MyAssays Ltd, East Sussex, UK) was used to calculate the aMT6s concentrations from a standard curve drawn based on 4 Parameter Logistics principles [44].

The urinary aMT6s concentrations were reported as uncorrected for creatinine (ng/mL) because detectability of its morning levels was uncertain. To the best of the authors’ knowledge, no local reference had reported on healthy individuals urinary aMT6s physiological range over time, and the impact of lighting on its concentrations. Studies highlighted daytime urinary aMT6s levels were very low [45], and the concentration of circulating melatonin in bodily fluids was often undetectable after 10am [26,46]. Interestingly, Graham et al. [47] reported a wider concentration range for the uncorrected urinary aMT6s than the creatinine-corrected samples between 11pm to 7am; suggesting higher chances of detectability with the uncorrected approach.

Besides, there were other concerns in measuring creatinine-corrected urinary aMT6s from the morning samples. It is an approach commonly used for predicting the peak nocturnal plasma melatonin levels [43,47], which was not the aim of the present study but recommended for further investigations. Caution is required in interpreting creatinine-corrected urinary aMT6s as urinary creatinine concentration is influenced by factors like ethnicity and body weight [43,48]. In fact, Graham et al. [47] reported the Spearman correlation between the log-transformed morning urinary aMT6s level uncorrected for creatinine with the log-transformed AUC of nocturnal plasma melatonin was [r = 0.69, p < 0.0001], which increased to [r = 0.76, p < 0.0001] when urinary aMT6s was corrected for creatinine. Both the uncorrected and corrected urinary aMT6s did show a significant, positive and moderately strong relationship [49] with the total amount of plasma melatonin secreted over the night. Therefore, the uncorrected approach was posited as more feasible in providing the preliminary empirical data required for this study.

#### Subjective alertness (psychological indicator for circadian system route)

Alertness was assessed using Karolinska Sleepiness Scale (KSS) [50], which had been validated against electroencephalography data [51]. Participants evaluated their actual alertness-sleepiness level during the past 5 minutes, under each luminous condition. The response options ranged from ‘extremely alert’ (1) to ‘fighting sleep’ (9).

#### Cognitive task performance (psychological indicator for circadian system route)

Sustained attention was objectively assessed using a computerized neurocognitive battery from Cogtest (Newark, DE). This battery is commonly used to measure the cognitive function in clinical trials. The Sustained Attention Test (SAT) measures the participant’s ability to withhold responses to unpredictable stimuli during a period of rapid and rhythmic response. Participants were instructed to click the respective mouse buttons for each of the 54 Non-Zero Condition (NZ) and Specific Condition (SP) stimuli. They were requested to focus, read the instructions carefully, and perform the SAT as quickly and accurately as possible under each luminous condition. The cognitive performance score (nos./sec) was calculated as:

$${\text{P}}_{\text{cog}}=\left[\left(\frac{\text{Total}\;\text{Correct}\;\text{Responses}\;\text{in}\;\text{NZ}\;\text{out}\;\text{of}\;54}{\text{Total}\;\text{Reaction}\;\text{Time}\;\text{for}\;\text{NZ}\;\text{Responses}}\right)+\left(\frac{\text{Total}\;\text{Correct}\;\text{Responses}\;\text{in}\;\text{SP}\;\text{out}\;\text{of}\;54}{\text{Total}\;\text{Reaction}\;\text{Time}\;\text{for}\;\text{SP}\;\text{Responses}}\right)\right]$$ (1)

#### Mood (psychological indicator for mood route)

Momentary mood ratings were evaluated using Positive and Negative Affect Schedule (PANAS) scale [52], with definitions for each adjective provided (computed Cronbach α = 0.947). The participants rated the extent each adjective described their current feelings under each luminous condition. Each adjective was rated based on a 5-point scale from ‘very slightly’ (1) to ‘extremely’ (5). The total score for positive affect (PA) and negative affect (NA) ranges from 10 to 50 each.

#### Visual task performance (psychological indicator for visual system route)

Visual performance for acuity and contrast was assessed using Freiburg Visual Acuity and Contrast Test (FrACT) [53]. This computer-based task required accurate and rapid recognition of each 36 Landolt ring’s orientation, under each luminous condition. For the acuity test, the stimuli came in different sizes and orientations; while for the contrast test, they came in different contrast with the background and orientations. Participants responded to each stimulus by clicking the matching keyboard cursor as quickly and accurately as possible. The acuity and contrast performance score (nos./sec) was calculated as:

$${\text{P}}_{\text{acuity}}=\frac{\text{Total}\;\text{correctly}\;\text{identified}\;\text{Landolt}\;\text{rings}\;\text{in}\;\text{Acuity}\;\text{out}\;\text{of}\;36\;\text{rings}}{\text{Total}\;\text{Reaction}\;\text{Time}\;\text{for}\;\text{Acuity}}$$ (2)

$${\text{P}}_{\text{contrast}}=\frac{\text{Total}\;\text{correctly}\;\text{identified}\;\text{Landolt}\;\text{rings}\;\text{in}\;\text{Contrast}\;\text{out}\;\text{of}\;36\;\text{rings}}{\text{Total}\;\text{Reaction}\;\text{Time}\;\text{for}\;\text{Contrast}}$$ (3)

#### Visual comfort assessment (psychological indicator for visual system route)

Visual comfort and general sensitivity under each luminous condition were evaluated using a modified version of Office Lighting Survey [54] and Lighting Belief Questionnaire [55]. This modified questionnaire consisted of 13 items, covering a mix scope of general, lighting-specific and minor health effect inquiries (computed Cronbach α = 0.816). A scoring system was designed, where the highest achievable score was +16 and lowest was -13 (S2 Appendix). Improved visual comfort was perceived when the total score moved closer towards the positive end, while worsened visual comfort was perceived if the total score moved towards the negative end.

### Statistical analyses

Each indicator was examined for normality. Urinary aMT6s and NA data had strong and positively skewed residual distribution [56], thus were log-transformed to attain satisfactory Skewness Index ± 3 [57]. Generalized Linear Mixed Model (GLMM, IBM SPSS, v.22) was performed to analyze the results of this triple nested data structure. Separate GLMM analyses were run for each measured indicator. The analyses took into account the hierarchical data structure, fixed effects, random effects, and covariates. Fig 2 illustrates the IPWI were repeatedly measured from participants who were exposed to different oscillations associated with a particular E_H_ light-setting; indicating measurements that were nested within successive levels of data hierarchy [58].

The factors for fixed effects included oscillation (the predetermined light-settings), time (TP_1_ and TP_2_) and oscillation*time (the interaction model to evaluate each light-setting’s immediate impact on the indicator over time). The interaction model was then tested for contrast, and the multiple comparisons were adjusted with sequential Bonferroni to reduce familywise Type1 error. For covariates, participant’s characteristics like ethnicity, sleep duration and staying up late status (yes or no response in the short questionnaire if he stayed awake beyond an estimated ± 30 minutes from his habitual sleep time) were considered due to its possible confounding effects. The 3 covariates were introduced one by one into the analysis and were omitted from the analysis if it increased the Akaike Information Criterion (AIC) value and did not contribute to any significant effect on the indicator (p > 0.05).

Random effects were considered to vary across the participants (Level: Individual). A better fit model was produced with random intercept and slope based on the AIC value. Oscillation was assigned as the random slope to consider the within-individual differences in the indicator due to lighting patterns. The participants represented a sample of the bigger population of university postgraduate students (young, dayshift working adults) from tropical Malaysia, while the oscillations were from a population of possible lighting configurations that could influence the circadian rhythm of the measured IPWI during a morning period.

GLMM’s output for each indicator provided the interaction effect results (F-statistics, Estimated Marginal Mean (EMM) for TP_1_ and TP_2_, the t-test, unstandardized coefficient (*B*) and confidence interval (CI)). These results enabled the comparison of the indicator’s immediate change over time in all the light-settings, and the immediate impact of each light-setting on the indicator in comparison to control. Testing the *B* estimate identified which light-setting differed significantly from control. The *B* estimated the magnitude and direction of each light-setting’s impact (*X*) on the indicator (*Y*). It reflected the light-setting’s group mean score of the indicator, which had been centered and rescaled to relate to the control’s hypothetical mean score set at value 0 [58]. Since there is no established formula as yet to calculate the composite index score for the 8 measured IPWI collectively; the abovementioned results were descriptively compared to recommend prospective light-settings for further investigations.

## Results and discussion

### Participants

All the 45 participants completed both the sessions from start to end. They consisted of different ethnic groups (48.9% Malays, 37.8% Chinese, 13.3% Indians), with mean (SD) age of 26.0 (3.1) years. Their chronotype were non-extreme morning or evening types with MEQ score of 53.69 (7.12). The participants from the 4 E_H_ light-settings represented a homogenous sample as they fulfilled the inclusion criteria, minimizing selection, maturation and regression threats [29]. Table 2 presents the descriptive statistics of the characteristics of participants in each E_H_ light-setting. Age and MEQ score (analyzed with 1-way ANOVA), and ethnicity (analyzed with Kruskal Wallis) showed no statistically significant difference across the 4 E_H_ light-settings. Bedtime prior to the experimental day, wake time on the experimental day, and sleep duration (analyzed with 1-way ANOVA) were also similar and showed no statistically significant difference across the light-settings.

**Table 2:** Characteristics of participants in each E_H_ light-setting.

	Constant Lighting		Dynamic Lighting						Overall
E_H_ light-setting	500-with-500 lx (JKR standard)		500-with-250 lx (low)		500-with-750 lx (high)		500-with-1000 lx (higher)		
Number of participants	n = 11 (Group A)		n = 11 (Group B)		n = 12 (Group C)		n = 11 (Group D)		n = 45
Age [mean (SD) years]	26.2 (2.3)		26.1 (3.4)		25.3 (3.1)		26.5 (3.6)		26.0 (3.1)
Ethnicity (%):									
Malay	54.5		54.5		41.7		45.5		48.9
Chinese	27.3		27.3		50.0		45.5		37.8
Indian	18.2		18.2		8.3		9.1		13.3
MEQ score [mean (SD)]	50.45 (6.50)		53.18 (8.57)		56.25 (5.19)		54.64 (7.54)		53.69 (7.12)
Light-setting (lx)	visit 1: 500 _constant_ 500 (con-trol)	visit 2: 500 _constant_ 500	visit 1: 500 _decreased to_ 250	visit 2: 250 _increased to_ 500	visit 1: 500 _increased to_ 750	visit 2: 750 _decreased to_ 500	visit 1: 500 _increased to_ 1000	visit 2: 1000 _decreased to_ 500	
Bedtime prior to the experimental day [mean (SD) pm]	10.51 (0.45)	11.02 (1.08)	10.43 (0.42)	10.54 (0.35)	11.18 (0.55)	11.11 (1.26)	11.21 (0.39)	11.47 (0.56)	11.08 (0.57)
Wake time on the experimental day [mean (SD) am]	7.05 (0.40)	6.57 (0.42)	6.54 (0.40)	7.11 (0.37)	6.53 (0.57)	7.05 (0.35)	7.24 (0.36)	7.24 (0.34)	7.06 (0.41)
Sleep duration [mean (SD) hours]	8.13 (0.45)	7.55 (1.18)	8.11 (0.36)	8.17 (0.23)	7.34 (0.45)	7.54 (1.39)	8.02 (0.37)	7.37 (1.02)	7.57 (0.58)

### Urinary aMT6s

**Fig 5:**
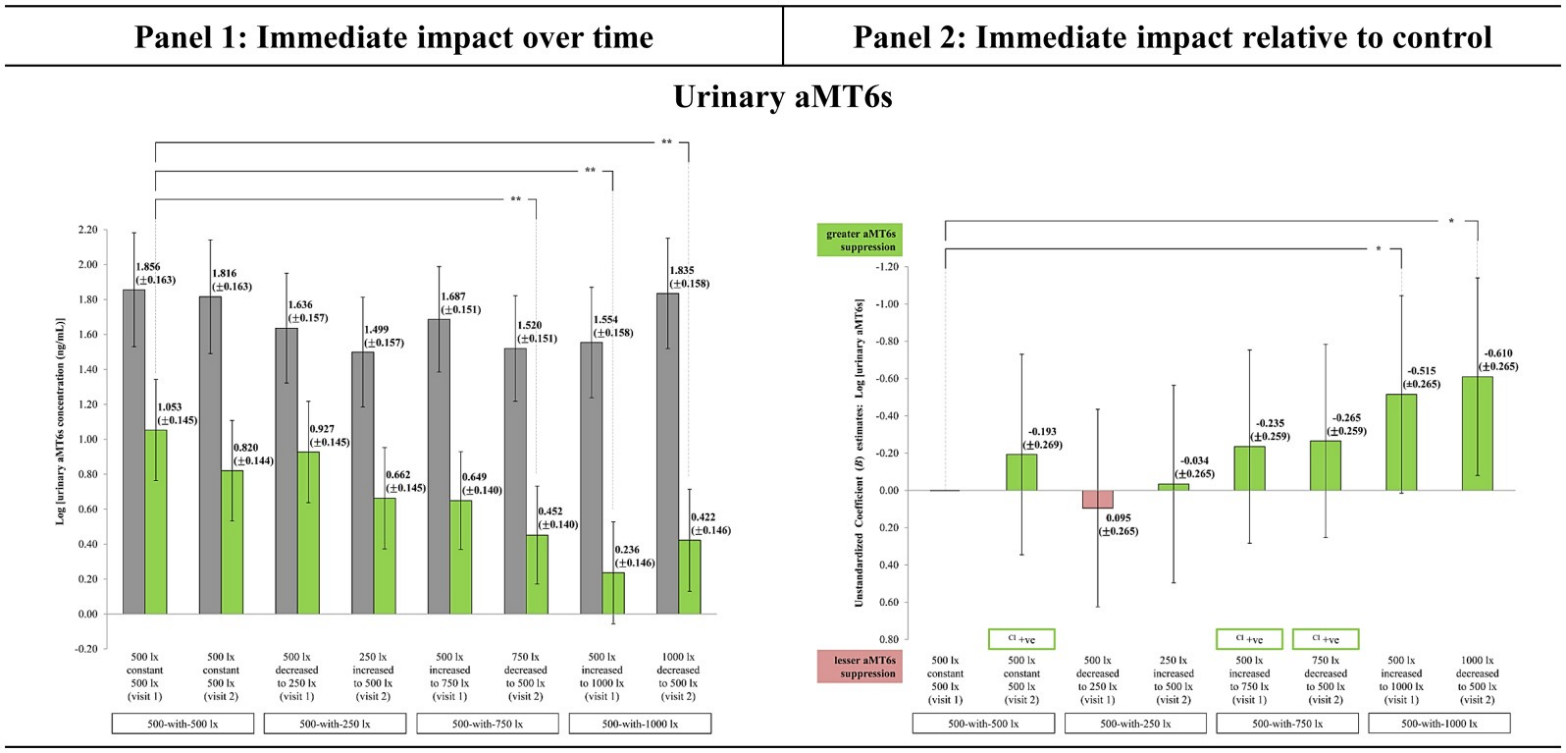
The immediate impact observed in Urinary aMT6s across the light-settings. (Panel 1) Immediate change over time in all light-settings: Bars represent Estimated Marginal Means (EMM) with Standard Errors (SE); whiskers 95% confidence interval (CI); *** p<0.001, ** p<0.01, * p<0.05, ^ p<0.06. Dark grey bar specifies EMM at TP_1_. Green bar specifies EMM at TP_2_ (supportive change). (Panel 2) Immediate impact of each light-setting relative to control: Bars represent Unstandardized Coefficient (*B*) with Standard Errors (SE); whiskers 95% CI; *** p<0.001, ** p<0.01, * p<0.05, ^ p<0.06. Green bar specifies light-setting as more supportive than control. Red bar specifies light-setting as less supportive than control. (S2 Fig) The chronological order of each light-setting’s EMM Δ for urinary aMT6s. It is recommended to be referred along with Fig 5 to improve the comprehension between panel 1 and 2. Light-setting with EMM Δ chronologically ordered above than the control (S2 Fig) corresponds with the direction of the *B* estimate bar as being more supportive than control (Fig 5, panel 2).

The fixed effects analysis result showed the interaction effect approached statistical significance [F(7,84) = 1.772, p < 0.06]. Each light-setting’s EMM concentration at TP_1_ (Fig 5, panel 1) was within the uncorrected urinary aMT6s physiological range (4.1 to 145.5 ng/mL) reported in Graham et al. [47]. All light-settings had significant immediate decline in urinary aMT6s concentration over the morning work period (p < 0.001). This supports the melatonin’s circadian profile, where morning concentrations gradually decline towards noon [11]. Post-exposure 1000_decreased to_500 significantly suppressed urinary aMT6s the most (reduction of 1.413 ng/mL), while 500_decreased to_250 the least (reduction of 0.709 ng/mL, half the effect of the former). As expected from literature, the ‘higher’ light-setting (brighter lighting) produced the greatest suppression than any other light-settings [20,59].

Moreover, the EMM concentration at TP_1_ was similar across the light-settings and was not significantly different when compared to control. This result strengthened the experimental study, where urinary aMT6s concentrations had no significant difference before any exposures were given to the participants. Significant difference was only observed after the 2-hour in-lab exposure to specific light-settings i.e. 1000_decreased to_500 [t(63) = -3.089; p = 0.009], 500_increased to_1000 [t(75) = -4.047; p = 0.001] and 750_decreased to_500 [t(74) = -3.046; p = 0.009], as shown in Fig 5, panel 1. The different declining rate observed across the light-settings indicated the in-lab ambient lighting influenced the suppression effect. Possible suppression effect due to retinal stimulation by the LED-backlit monitor was regarded as less influential because the participants were exposed to monitors that had similar luminance and had been controlled.

At present, only dynamic lighting 500-with-1000 in decreasing and increasing oscillation differed significantly from control (Fig 5, panel 2). The 1000_decreased to_500 [*B* = -0.610; SE = 0.265; p = 0.012] had the greatest suppression effect, followed by 500_increased to_1000 [*B* = -0.515; SE = 0.265; p = 0.028] relative to control. Both these configurations significantly suppressed the morning melatonin to low levels (almost twice more the impact of 500-with-750). This result supports the studies from Japan [21,40] which observed bright light (BL) contributed towards significantly greater magnitude of morning melatonin suppression than dim light (DL), contrasting studies from the European context who were unable to detect any significant difference in its daytime declining rate between BL versus DL [22,24]. The greater magnitude of suppression observed in the decreasing oscillation supported the protocol’s lighting pattern for a morning period.

There are spill-over benefits with greater melatonin suppression in the mornings. BL that suppressed melatonin to low levels during the day, in a rebound response resulted in greater amounts of nocturnal secretion [21,60]. This consequently improved sleep quality at night [61] and daytime alertness the following day [35,40]. Whether these reported beneficial impacts (experienced by samples from seasonal climate) could replicate for a Malaysian sample experiencing tropical climate is subjected to future investigations.

### Subjective alertness

The fixed effects analysis result showed the interaction effect approached statistical significance [F(7,83) = 1.781, p < 0.06]. There was no significant difference in the EMM alertness score at TP_1_ across the light-settings that started with 500 lx during visit 1. This result strengthened the experimental study, as participants were comparable at the 500 lx exposure in each of the 4 E_H_ light-settings (‘visit 1: 500_constant_500’, 500_decreased to_250, 500_increased to_750, and 500_increased to_1000 lx).

**Fig 6:**
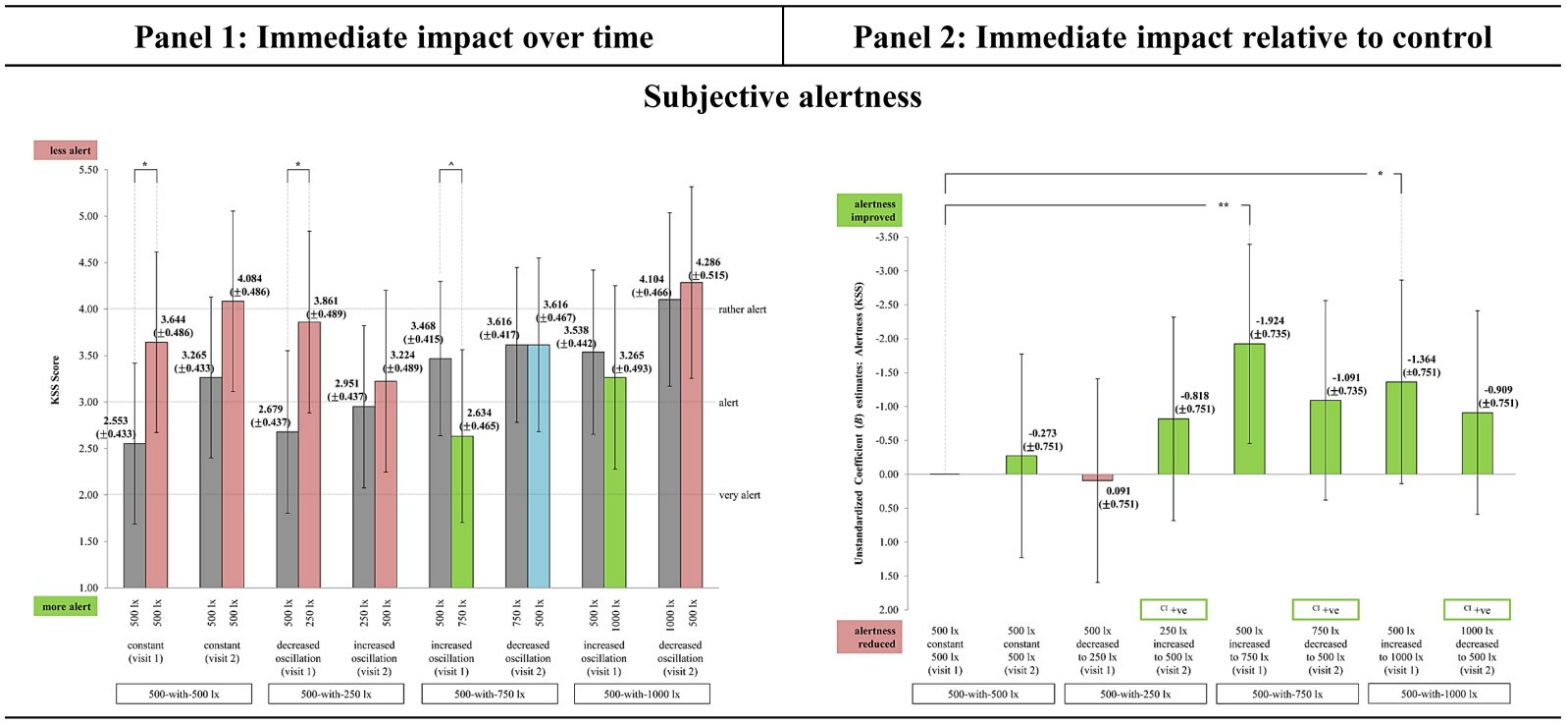
The immediate impact observed in Subjective alertness across the light-settings. (Panel 1) Immediate change over time in all light-settings: Bars represent Estimated Marginal Means (EMM) with Standard Errors (SE); whiskers 95% confidence interval (CI); *** p<0.001, ** p<0.01, * p<0.05, ^ p<0.06. Dark grey bar specifies EMM at TP_1_. Green bar specifies EMM at TP_2_ (supportive change). Red bar specifies EMM at TP_2_ (unsupportive change). Light blue bar specifies EMM at TP_2_ (no change). (Panel 2) Immediate impact of each light-setting relative to control: Bars represent Unstandardized Coefficient (*B*) with Standard Errors (SE); whiskers 95% CI; *** p<0.001, ** p<0.01, * p<0.05, ^ p<0.06. Green bar specifies light-setting as more supportive than control. Red bar specifies light-setting as less supportive than control. (S2 Fig) The chronological order of each light-setting’s EMM Δ for subjective alertness. It is recommended to be referred along with Fig 6 to improve the comprehension between panel 1 and 2. Light-setting with EMM Δ chronologically ordered above than the control (S2 Fig) corresponds with the direction of the *B* estimate bar as being more supportive than control (Fig 6, panel 2).

All light-settings had EMM alertness score between ‘very alert’ to ‘rather alert’ during TP_1_ and TP_2_, indicating the participants were on the alert side of the scale [50]. Despite so, all light-settings (except 750_decreased to_500) had immediate fluctuations in morning alertness over time (Fig 6, panel 1). Light-setting 500_increased to_750 approached statistical significance [t(83) = -1.640; p < 0.06] in improving morning alertness immediately, while 500_decreased to_250 [t(83) = 2.226; p = 0.015] and control [t(83) = 2.055; p = 0.022] resulted in a significant reduction.

The different directions of immediate change in morning alertness across the light-settings indicated a trend, which impact was likely influenced by the incorporated E_H_ level and oscillation, with 500 lx appearing to be a baseline level in deciding the direction of change. Dynamic lighting in increasing oscillation (500_increased to_750 and 500_increased to_1000) further improved alertness, suggesting lighting that turned brighter from 500 lx only supported morning alertness. Decreasing the E_H_ levels towards 500 lx from a brighter E_H_ level (750 and 1000 lx), and using E_H_ ≤ 500 lx (250_increased to_500 and 500_decreased to_250) could have lacked stimuli to activate alertness in the tropics, despite being dynamic interventions with white LED (6500 K). The reduction in alertness in constant lighting was likely due to boredom and lack of varying light stimulus over time.

These results provided additional input on the acute improvement in morning alertness with bright light in comparison to Huiberts et al. [8], Smolders et al. [9], and Souman et al. [62]. This study observed incorporating brighter light ranges (like 500-with-750 and 500-with-1000 lx) may not always contribute towards improved morning alertness, as the configured dynamic lighting pattern influenced the morning boosting effect. At present, relative to control, light-settings 500_increased to_750 [*B* = -1.924; SE = 0.735; p = 0.006] contributed towards the greatest improvement in morning alertness, followed by 500_increased to_1000 [*B* = -1.364; SE = 0.751; p = 0.037]. Both these configurations indicated to be more stimulating than the control lighting in improving morning alertness in WOPW in Malaysia (Fig 6, panel 2).

### Mood

The fixed effects analysis result showed the interaction effect for PA [F(7,90) = 4.098, p = 0.001] and NA [F(7,84) = 2.712, p = 0.007] were statistically significant. There was no significant difference in the EMM PA, and NA score at TP_1_ across the light-settings that started with 500 lx during visit 1. This result strengthened the experimental study, as participants were comparable at the 500 lx exposure in each of the 4 E_H_ light-settings (‘visit 1: 500_constant_500’, 500_decreased to_250, 500_increased to_750, and 500_increased to_1000 lx).

**Fig 7:**
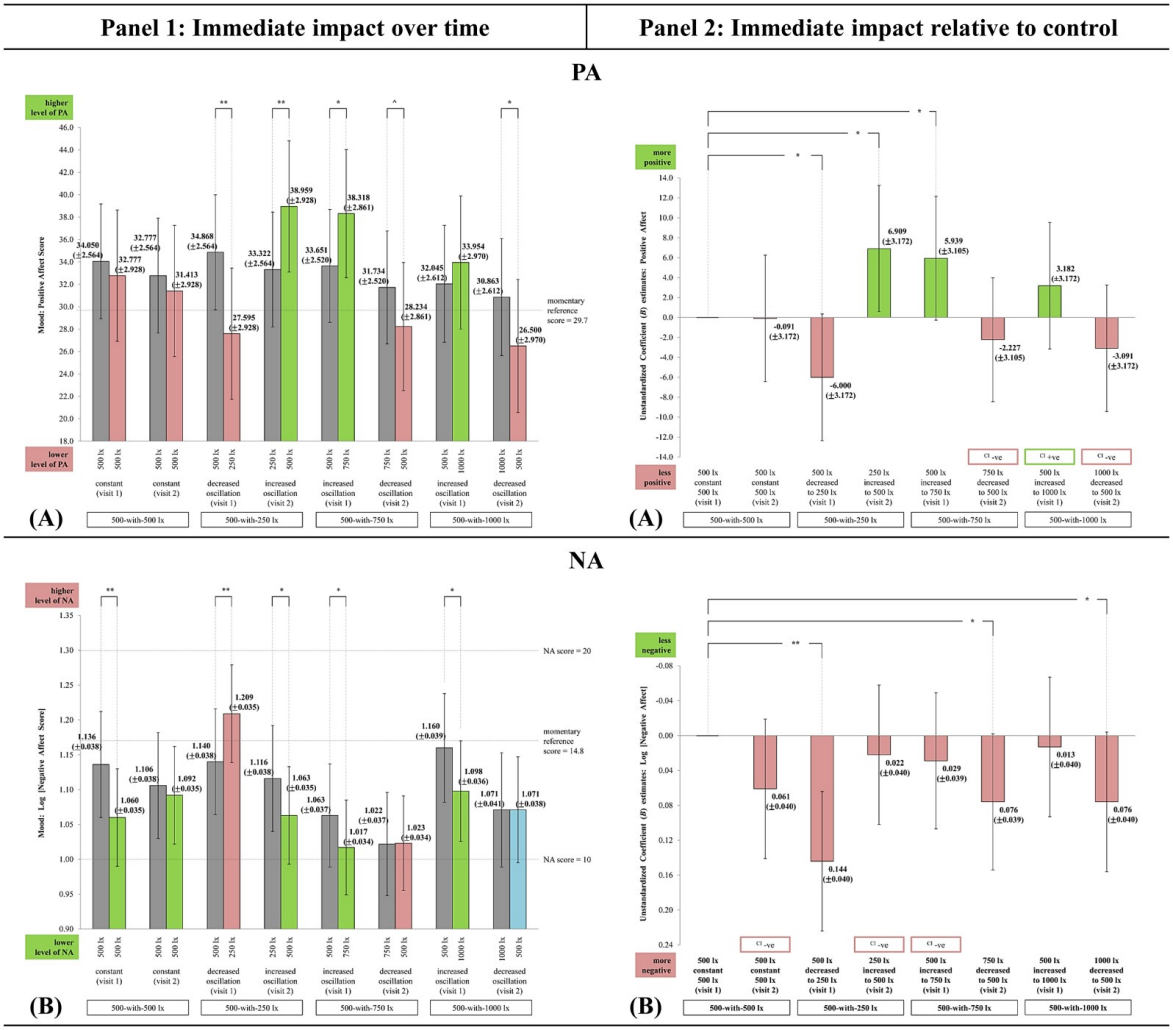
The immediate impact observed in PA and NA across the light-settings. (A) PA. (B) NA. (7A–7B, panel 1) Immediate change over time in all light-settings: Bars represent Estimated Marginal Means (EMM) with Standard Errors (SE); whiskers 95% confidence interval (CI); *** p<0.001, ** p<0.01, * p<0.05, ^ p<0.06. Dark grey bar specifies EMM at TP_1_. Green bar specifies EMM at TP_2_ (supportive change). Red bar specifies EMM at TP_2_ (unsupportive change). Light blue bar specifies EMM at TP_2_ (no change). (7A–7B, panel 2) Immediate impact of each light-setting relative to control: Bars represent Unstandardized Coefficient (*B*) with Standard Errors (SE); whiskers 95% CI; *** p<0.001, ** p<0.01, * p<0.05, ^ p<0.06. Green bar specifies light-setting as more supportive than control. Red bar specifies light-setting as less supportive than control. (S2 Fig) The chronological order of each light-setting’s EMM Δ for PA and NA. It is recommended to be referred along with Figs 7A–7B to improve the comprehension between panel 1 and 2. Light-setting with EMM Δ chronologically ordered above than the control (S2 Fig) corresponds with the direction of the *B* estimate bar as being more supportive than control (Figs 7A–7B, panel 2).

All light-settings started with higher PA and lower NA scores than the respective momentary reference score reported by Watson et al. [52]. This indicated the participants were in a state of high energy, full concentration, pleasurable engagement (high PA), and calmness and serenity (low NA) [52,63]. Despite so, all the light-settings had immediate fluctuations in PA and NA scores over time, except 1000_decreased to_500 for NA (Fig 7A and 7B, panel 1). The different directions of immediate change in mood across the light-settings indicated a trend, which impact was likely influenced by the oscillation effect.

1. Dynamic lighting in increasing oscillation further increased the PA and decreased the NA scores over time, hence improved the participants’ state of high energy, full concentration, pleasurable engagement, calmness, and serenity. Light-settings 250_increased to_500 was better for improving morning PA [PA: t(90) = 2.513, p = 0.007; NA: t(84) = -1.869, p = 0.033], followed by 500_increased to_750 [PA: t(90) = 2.173, p = 0.016; NA: t(84) = -1.687, p = 0.048], while 500_increased to_1000 was better in immediately decreasing morning NA [PA: t(90) = 0.851, n.s.; NA: t(84) = -2.184, p = 0.016]. Even though this result supports most literature that exposure to more light improved daytime mood and vitality; all the same it provided additional input on the impact of dynamic lighting on morning mood in comparison to Vallenduuk [14]. This tropical study revealed both PA and NA fluctuated, as opposed to the seasonal climate study which observed only PA significantly improved over time (between 9.30am to 11am), while NA remained steady.
2. Dynamic lighting in decreasing oscillation decreased the PA to below the momentary reference score, and tended in increasing NA scores, resulting towards a probable state of lethargy/depression [52,63]. Light-setting 500_decreased to_250 significantly decreased PA and increased NA the most [PA: t(90) = -3.243, p = 0.001; NA: t(84) = 2.412, p = 0.009]. Decrease in PA was also observed in 750_decreased to_500 [t(90) = -1.630; p < 0.06] and 1000_decreased to_500 [t(90) = -1.946; p = 0.028], but they both did not impact NA much.
3. Constant lighting decreased both PA and NA over time. The decrease in PA scores over time was insignificant and lesser than the dynamic lighting in decreasing oscillation. However, the control had the greatest impact in decreasing morning NA than any other light-settings [t(84) = -2.645, p = 0.005]. This observation differed from studies that experienced seasonal climate. From the Netherlands, Vallenduuk [14] observed both PA and NA increased when exposed to constant lighting (E_H_ at desk plane = 1200 lx); while from China, Gou et al. [64] observed PA decreased and NA increased when exposed to constant lighting (E_H_ at desk plane = 257.61 lx).

The different direction of mood changes found between this study and those reported in [14,64,65] could likely be due to the difference between tropical and seasonal climate studies (socio-cultural preference), and the adopted E_H_ levels. At present, in comparison to control, dynamic lighting in increasing oscillation was observed more beneficial and supportive in increasing PA and contributing towards a lesser impact in increasing NA than any other light-settings in Malaysia (Fig 7A and 7B, panel 2). The 250_increased to_500 had the greatest impact in increasing morning PA [*B* = 6.909; SE = 3.172; p = 0.016] relative to control.

Dynamic lighting in decreasing oscillation and follow-up constant lighting were observed less supportive than control as they decreased PA and increased NA (at least twice greater an impact compared to dynamic lighting in increasing oscillation). Light-setting 500_decreased to_250 was the least supportive as it significantly decreased PA [*B* = -6.000; SE = 3.172; p = 0.031] and increased morning NA [*B* = 0.144; SE = 0.040; p = 0.001] relative to control. This result provided additional input to expand the findings of Canazei et al. [15] and Vallenduuk [14] that not all, but only specific dynamic lighting configurations functioned as a stronger mood enhancer than constant lighting.

### P_cog_, P_acuity_, and P_contrast_ task performance

The fixed effects analyses results showed the interaction effect for P_cog_ was statistically significant [F(7,82) = 4.967, p < 0.001], while not for P_acuity_ and P_contrast_. There was no significant difference in the EMM P_cog_, P_acuity_, and P_contrast_ score at TP_1_ across the light-settings that started with 500 lx during visit 1. This result strengthened the experimental study, as participants were comparable at the 500 lx exposure in each of the 4 E_H_ light-settings (‘visit 1: 500_constant_500’, 500_decreased to_250, 500_increased to_750, and 500_increased to_1000 lx).

**Fig 8:**
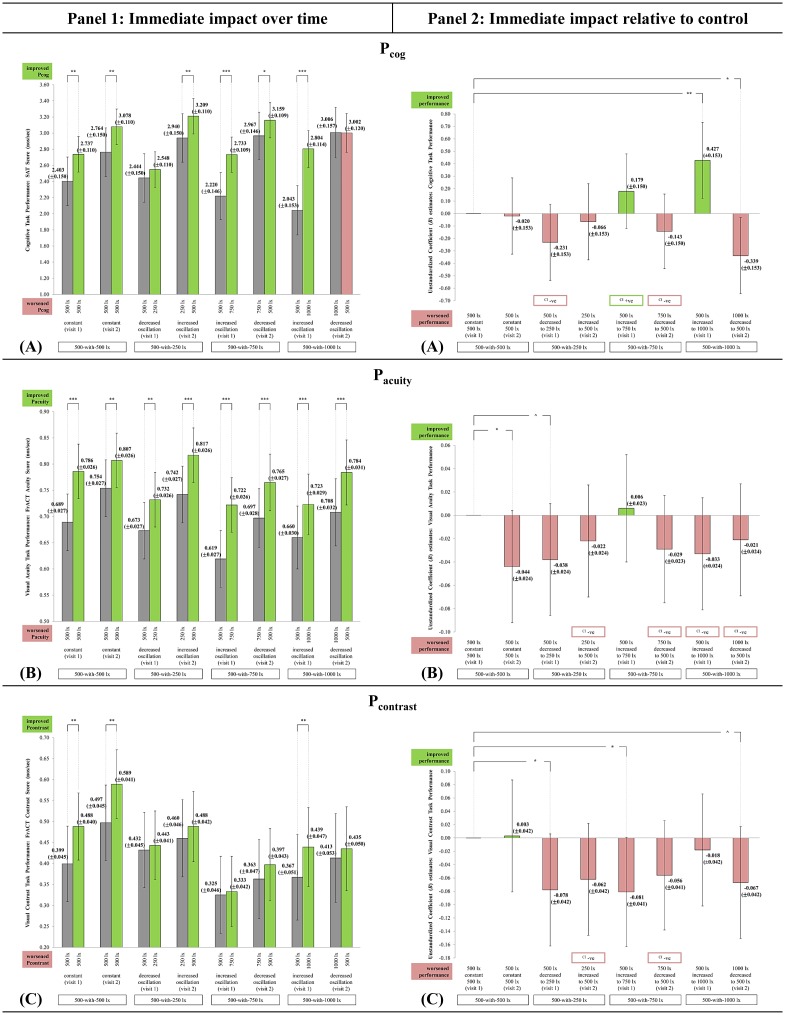
The immediate impact observed in P_cog_, P_acuity_, and P_contrast_ across the light-settings. (A) P_cog_. (B) P_acuity_. (C) P_contrast_. (8A–8C, panel 1) Immediate change over time in all light-settings: Bars represent Estimated Marginal Means (EMM) with Standard Errors (SE); whiskers 95% confidence interval (CI); *** p<0.001, ** p<0.01, * p<0.05, ^ p<0.06. Dark grey bar specifies EMM at TP_1_. Green bar specifies EMM at TP_2_ (supportive change). Red bar specifies EMM at TP_2_ (unsupportive change). (8A–8C, panel 2) Immediate impact of each light-setting relative to control: Bars represent Unstandardized Coefficient (*B*) with Standard Errors (SE); whiskers 95% CI; *** p<0.001, ** p<0.01, * p<0.05, ^ p<0.06. Green bar specifies light-setting as more supportive than control. Red bar specifies light-setting as less supportive than control. (S2 Fig) The chronological order of each light-setting’s EMM Δ for P_cog_, P_acuity_, and P_contrast_. It is recommended to be referred along with Figs 8A–8C to improve the comprehension between panel 1 and 2. Light-setting with EMM Δ chronologically ordered above than the control (S2 Fig) corresponds with the direction of the *B* estimate bar as being more supportive than control (Figs 8A–8C, panel 2).

There were immediate changes in P_cog_, P_acuity_, and P_contrast_ scores over time in all the light-settings (Fig 8A–8C, panel 1). The immediate improvement in the task performance during visit 1 in all the 4 E_H_ light-settings and with higher achievement scores during visit 2 suggest possibilities of:

1. Practice effect. The participants could have got familiar with the type of tasks, making them psychologically prepared for the rapid, random and unpredictable stimuli. Improvement in cognitive and visual task performance over time due to learning effect was also evidenced by Boyce et al. [66] and Vallenduuk [14].
2. Support from the blue-enriched characteristic of white LED (6500 K) lamp, but this is subjected to further investigations. Literature evidenced using higher CCT lamps improved daytime concentration and performance [16,67] and induced smaller pupil size which promoted good vision for task performance [17,41,68]. Higher CCT LED lighting also resulted in faster cognitive processing speed and reaction times in identifying symbol and recognizing color [32,69].

The improvement for morning P_cog_ was most in 500_increased to_1000 [t(82) = 7.030; p < 0.001], while 1000_decreased to_500 had a slight insignificant decline despite its high scores. For morning P_acuity_, improvement was most in 500_increased to_750 [t(81) = 6.434; p < 0.001], and least in ‘visit 2: 500_constant_500’ [t(81) = 3.179; p = 0.001]. The reverse was observed for morning P_contrast_, where improvement was most in ‘visit 2: 500_constant_500’ [t(79) = 3.100; p = 0.002] and least in 500_increased to_750 lx (n.s).

At present, dynamic lighting in increasing oscillation, specifically 500_increased to_1000 [*B* = 0.427; SE = 0.153; p = 0.004] and 500_increased to_750 [*B* = 0.179; SE = 0.150; n.s.] was observed as more beneficial and supportive than control in improving morning P_cog_ in WOPW in Malaysia (Fig 8A, panel 2). For P_acuity_ and P_contrast_, most of the dynamic light-settings were less supportive than control (Fig 8B and 8C, panel 2). Hence, short-term exposure to constant lighting was recommended as more supportive for morning P_acuity_ and P_contrast_ in WOPW in Malaysia, possibly due to lesser visual distraction. However, caution is raised on follow-up exposure to constant lighting because it significantly lessened P_acuity_ improvement.

### Visual comfort assessment

The fixed effects analysis result showed the interaction effect for visual comfort was statistically significant [F(7,90) = 6.821, p < 0.001]. There was no significant difference in the EMM visual comfort score at TP_1_ across the light-settings that started with 500 lx during visit 1. This result strengthened the experimental study, as participants were comparable at the 500 lx exposure in each of the 4 E_H_ light-settings (‘visit 1: 500_constant_500’, 500_decreased to_250, 500_increased to_750, and 500_increased to_1000 lx).

**Fig 9:**
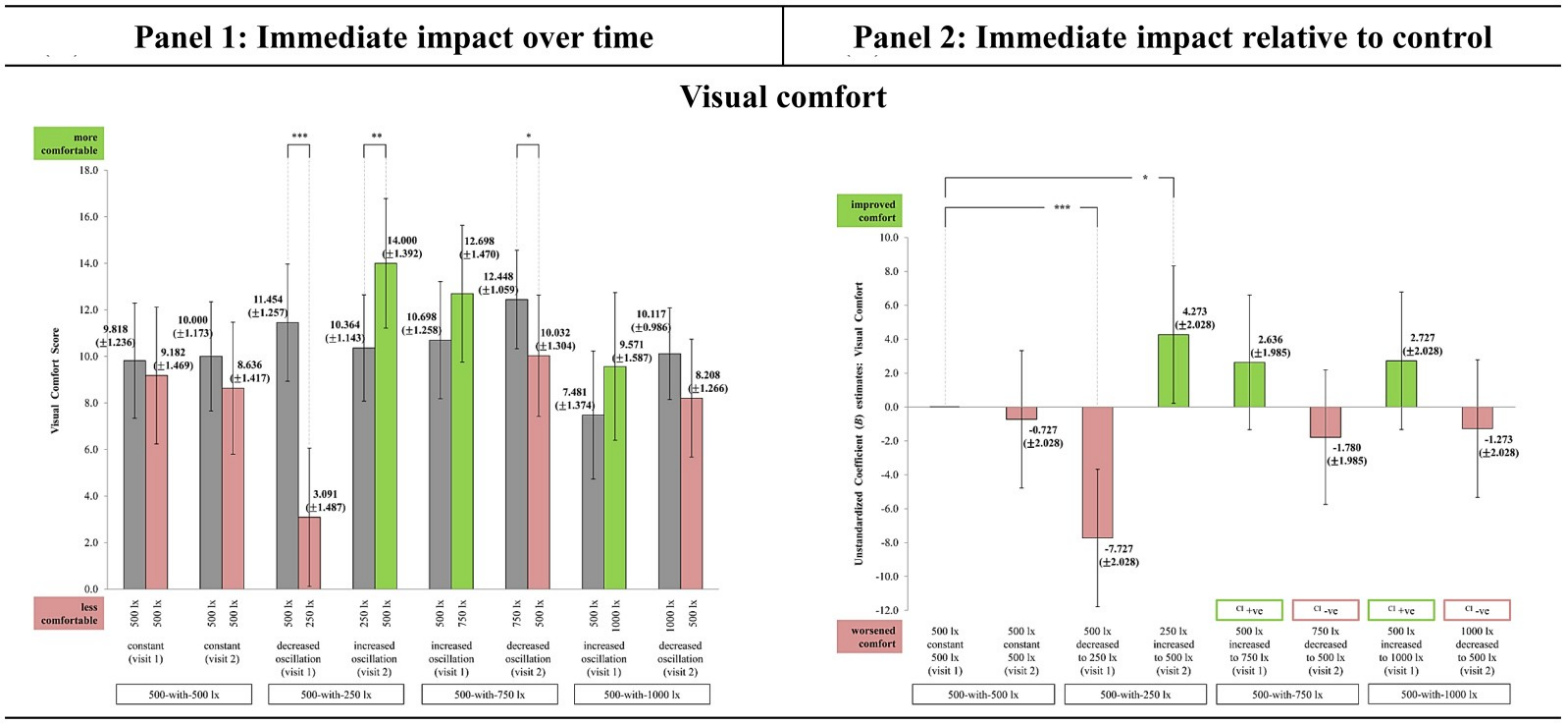
The immediate impact observed in Visual comfort across the light-settings. (Panel 1) Immediate change over time in all light-settings: Bars represent Estimated Marginal Means (EMM) with Standard Errors (SE); whiskers 95% confidence interval (CI); *** p<0.001, ** p<0.01, * p<0.05, ^ p<0.06. Dark grey bar specifies EMM at TP_1_. Green bar specifies EMM at TP_2_ (supportive change). Red bar specifies EMM at TP_2_ (unsupportive change). (Panel 2) Immediate impact of each light-setting relative to control: Bars represent Unstandardized Coefficient (*B*) with Standard Errors (SE); whiskers 95% CI; *** p<0.001, ** p<0.01, * p<0.05, ^ p<0.06. Green bar specifies light-setting as more supportive than control. Red bar specifies light-setting as less supportive than control. (S2 Fig) The chronological order of each light-setting’s EMM Δ for visual comfort. It is recommended to be referred along with Fig 9 to improve the comprehension between panel 1 and 2. Light-setting with EMM Δ chronologically ordered above than the control (S2 Fig) corresponds with the direction of the *B* estimate bar as being more supportive than control (Fig 9, panel 2).

All light-settings had EMM visual comfort score that ranged from 3.091 to 14.000 during TP_1_ and TP_2_. This provided evidence that the ambient lighting did not cause visual discomfort because the scores were on the positive end of the scale, indicating the participants’ were in a comfortable state. Despite so, all the light-settings had immediate fluctuations in visual comfort scores over time (Fig 9, panel 1). The different directions of immediate change indicated a trend likely influenced by the oscillation effect. Alike PA, dynamic lighting in increasing oscillation further improved visual comfort with 250_increased to_500 [t(90) = 2.536; p = 0.007] showed the greatest improvement. In contrast, constant lighting and dynamic lighting in decreasing oscillation further reduced visual comfort with 500_decreased to_250 [t(90) = -5.832; p < 0.001] showed the greatest reduction.

At present, only dynamic lighting in increasing oscillation was observed as more supportive than the control in improving morning visual comfort, while all the other light-settings were observed as less supportive (Fig 9, panel 2). The improvement in visual comfort with increasing E_H_ levels in the morning supports Begemann et al. [5]. It indicated workplace lighting which mimics the natural daylight cycle had a more beneficial impact than constant lighting. Light-setting 250_increased to_500 [*B* = 4.273; SE = 2.028; p = 0.019] was most supportive relative to control, while 500_decreased to_250 [*B* = -7.727; SE = 2.028; p < 0.001] was the least. The ‘low’ E_H_ light-setting had the most significant impact possibly due to the distinct difference in the visual appraisal, i.e., between a dim (250 lx) and brighter (500 lx) E_H_ level.

## Conclusion and recommendations

The overhead white LED (6500 K) ambient lighting in the WOPW immediately impacted the measured IPWI either towards a supportive or unsupportive change over time (S3 Table). Incorporating constant lighting (JKR standard) supported 5 of the 8 indicators in the morning, while dynamic lighting in increasing oscillation (specifically 500_increased to_750 and 500_increased to_1000 lx) supported all the 8 indicators in the direction needed for a morning boost during the peak morning work period.

In comparison to control, there were light-settings with either clinically inferred beneficial or non-beneficial impacts (S4 Table). According to Page [70], a wide CI that remained relatively large on the supportive end (common for small sample size) suggest the confidence and possibilities of a clinically beneficial impact despite their statistically insignificant result. Hence, the descriptive comparison revealed follow-up constant lighting only supported 1 of the 8 measured indicators (12.5%). Interestingly, light-settings 500_increased to_750 and 500_increased to_1000 lx supported most of the measured indicators (5 of 8 = 62.5%) from the 3 routes, where they suppressed urinary aMT6s, and improved alertness, P_cog_, PA and visual comfort better than control.

This study provided evidence that applying supportive, dynamic lighting in increasing oscillation (specifically 500_increased to_750 and 500_increased to_1000 lx) resulted towards a better morning boosting effect, with an additional 50% support on the IPWI compared to the control constant lighting. The dynamic lighting in decreasing oscillation revealed to be non-supportive of the morning boosting effect because they negatively impacted 6 of the 8 measured indicators. Therefore, not all, but only 2 specific dynamic lighting configurations in increasing oscillation were more supportive of the morning boost. These findings present the feasibility of supportive, dynamic architectural lighting acting as an environmental therapeutic solution in supporting IPWI in WOPW in tropical Malaysia.

**Fig 10:**
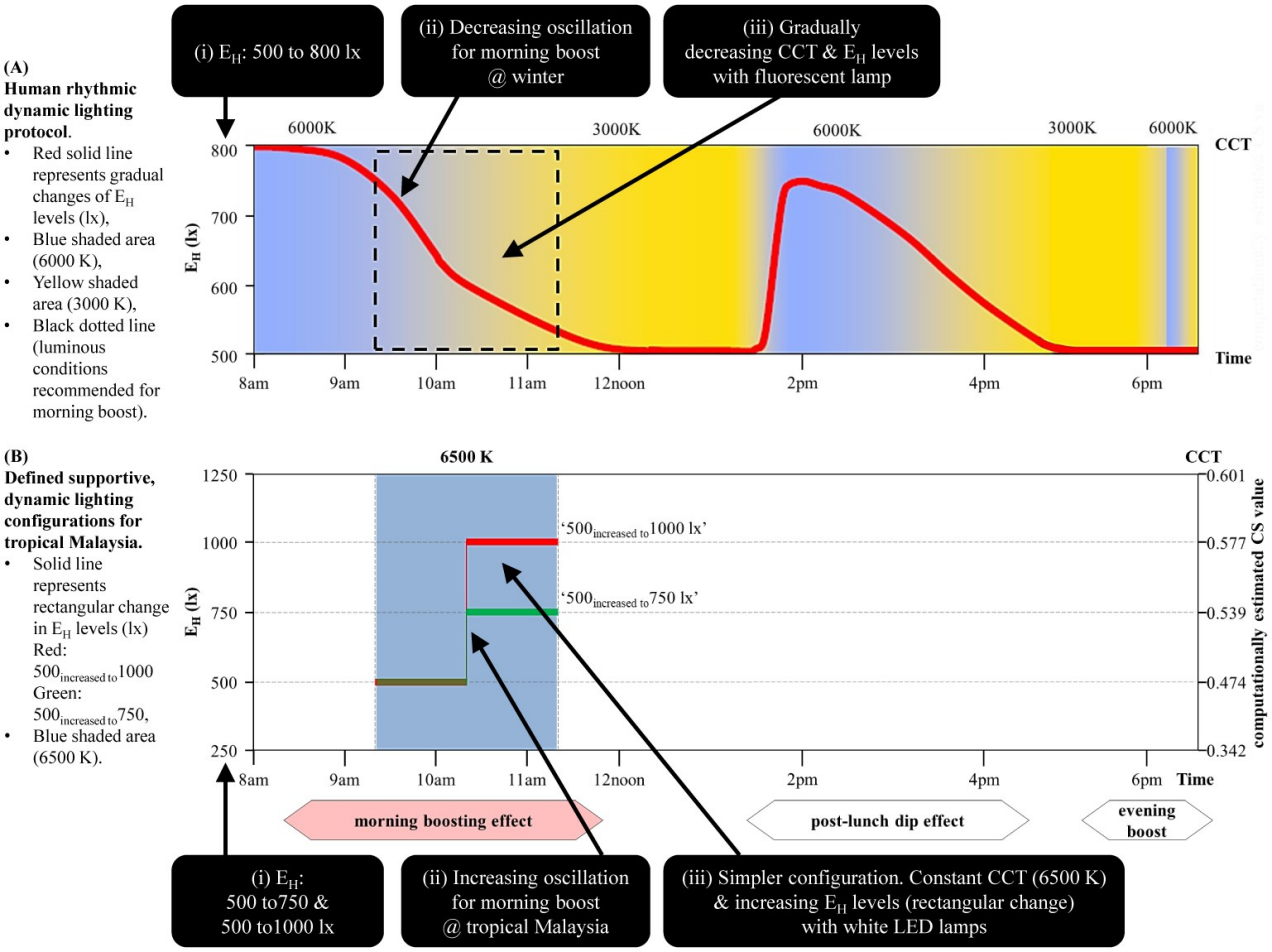
Key differences in the luminous conditions for boosting effect during the peak morning work period. (A) Human rhythmic dynamic lighting protocol by van Bommel [11,12]. (B) Defined supportive, dynamic lighting configurations for tropical Malaysia.

With that, this study recommends dynamic, overhead white LED (6500 K) ambient lighting with configurations 500_increased to_750 and 500_increased to_1000 lx for further and larger sample size investigations, to determine the better supportive one for the morning boosting effect in Malaysia. Both these prospective configurations established the preliminary groundwork in defining the supportive, dynamic lighting configurations for tropical Malaysia. They revealed 3 key differences from the human rhythmic protocol (Fig 10).

1. The E_H_ levels of ‘500 to 750 lx’ and ‘500 to 1000 lx’ were observed more beneficial for the morning boosting effect in tropical Malaysia. These E_H_ levels were almost similar and higher compared to the ‘500 to 800 lx’ specified by the protocol. These brighter lighting configurations support Gou et al. [71] who reported incorporating higher illuminance levels has a restorative measure to create stimulating workplace that was favorable for wellbeing, productivity, and at the same time not considered as uncomfortable.
2. The increasing oscillation was observed more beneficial for the morning boost in tropical Malaysia. This lighting pattern was in reverse to the protocol’s decreasing oscillation for the morning boosting effect during winter.
3. Suggesting a possible simpler configuration for the morning boosting effect in tropical Malaysia, where maintaining a constant CCT (6500 K) and increasing the E_H_ levels (in a rectangular change) with white LED lamps were itself effective. This was an alternative to the protocol’s gradually decreasing CCT and E_H_ levels with fluorescent lamps. The simpler configuration (constant 6500 K in the morning) minimizes too many variations in the lighting conditions that could lead to confusion, distress [17], and distract the concentration needed for cognitive performance [71]. Although Smolders & de Kort [72] from the Netherlands reported no significant activating effects on the non-image-forming responses during regular working hours, except for higher vitality in the morning with the 6000 K condition, studies from the Asian contexts have indicated preference and beneficial impacts with the 6500 K CCT (S1 Appendix). Moreover, using LED lighting that provides a bluish color ambiance in WOPW supports not only the physiological response but psychological behavior too. A study from China reported such ambiance colored lighting was most preferred and created a more sensory, pleasing, relaxing workplace as it supplemented the missing bluish tinge of natural daylight [73]. Besides that, dynamically changing the E_H_ levels could provide an energy-efficient solution as workplace would not need to maintain higher E_H_ levels (≥ 1000 lx) over the morning work period. Savings in operational-cum-maintenance cost is expected with the usage of LED lamps [31]. However, whether these potential advantages could be reflected in real-life application is subjected to further investigations.

Since the dynamic architectural lighting for the tropics requires different configurations from that recommended by the protocol benchmarked based on seasonal climate requirements; more local multidisciplinary research is encouraged to provide additional and unequivocal empirical evidence to continue defining the supportive, dynamic architectural lighting for the tropics. Expanding the investigations to other times of the day would enable the development of an optimal dynamic lighting curve to support IPWI in WOPW in Malaysia. Incorporating detailed corrected and uncorrected urinary aMT6s analyses could establish a local normative data on its daily profile among healthy individuals for research merit comparisons. Extending investigations to other physiological indicators (core body temperature, cortisol, EEG brain waves, etc.) could provide more inputs on the IPWI. Investigating the impact on females and older age working adults are also necessary to optimize organizational productivity. These studies would support the vision of Malaysia’s smart city development, which prioritizes improving individuals’ wellbeing and human capital performance for sustainable economic growth.

## Limitations and strengths

Limitations. This study interpreted the immediate impact on the measured IPWI as a result of a 2-hour morning light exposure in a WOPW; hence generalization to other times of the day is limited. The sample consisted of male participants thus the results may be gender specific. The results of the psychological indicators may be influenced by Hawthorne effect, where participants’ behavior could have been influenced by being observed, which may have led to bias in responses. The urinary aMT6s were assayed with single detection and reported as uncorrected for creatinine; however, its limitations are believed to cause minimal bias to the results as explained in the urinary aMT6s subsection.

Strengths. Each participant was not put through the 4 E_H_ light-settings to minimize possible boredom/practice effect and personal pre-test biases influencing the results of a repeated measure design. Although the 4 E_H_ light-settings had its own randomly allocated participants (different subject between-groups) which makes comparison less powerful, having the participants within each E_H_ light-setting exposed to the different oscillations scheduled on 2 separate days in a counterbalanced order (repeated measures within-group) makes it a more valid design. Proper randomization and maintenance of blinding also strengthened the validity of this study. The results reflected the immediate impact of the light-settings on the IPWI during the peak morning work period, mimicking real-life situations and natural conditions.

With no local dynamic lighting configurations defined yet for the tropics, this study pioneered identifying dynamic lighting configurations that were more supportive of the morning boosting effect than the control constant lighting. Its initial local data established the preliminary groundwork in defining supportive, dynamic architectural lighting to support IPWI in WOPW in tropical Malaysia. Arousal and visual appraisal effects could have played a more prominent role in the morning boosting effect, alongside with the greater morning urinary aMT6s suppression impact. The preliminary empirical data on urinary aMT6s creates awareness on prospective research area in lighting and the circadian system for the tropics.

## Supporting information

S1 Table List of selected faculties/institution from Universiti Putra Malaysia. (DOCX)

S2 Table Researcher’s reference list. Information on the scheduled experimental sessions (2 separate dates) and 12 IDs randomly assigned to each of the 4 E_H_ light-setting.
(DOCX)

S3 Table Summary of the light-setting’s immediate impact over time on the measured IPWI. (DOCX)

S4 Table Summary of the light-setting’s immediate impact relative to control for the measured IPWI. (DOCX)

S1 Fig False color distribution of the 4 E_H_ levels (lx). (TIF)

S2 Fig The chronological order of each light-setting’s EMM Δ for each measured IPWI. (TIF)

S1 Appendix Specifications of the white LED lamp. (DOCX)

S2 Appendix Overview of the visual comfort assessment and its scoring. (DOCX)
