# Correction: Morning boost on individuals' psychophysiological wellbeing indicators with supportive, dynamic lighting in windowless open-plan workplace in Malaysia

**DOI:** 10.1371/journal.pone.0210190

**Published:** 2018-12-28

**Authors:** The PLOS ONE Staff

## Notice of Republication

This article was republished on December 17, 2018 to correct for Unicode character errors introduced during the typesetting process. The publisher apologizes for the errors. Please download this article again to view the correct version. The originally published, uncorrected article and the republished, corrected article are provided here for reference.

## Supporting information

S1 File Originally published, uncorrected article. (PDF)

S2 File Republished, corrected article. (PDF)
