# Supplementary material for: Morning boost on individuals’ psychophysiological wellbeing indicators with supportive, dynamic lighting in windowless open-plan workplace in Malaysia
Source: PLoS One. 2018 Nov 29;13(11):e0207488. doi: 10.1371/journal.pone.0207488 (PMC6264480; doi:10.1371/journal.pone.0207488)
Supplement: S1 Table — (DOCX) [file pone.0207488.s001.docx]

**S1 Table. List of selected faculties/institution from Universiti Putra Malaysia.**

| **Faculties/institution selected** | **Faculties excluded** |
| --- | --- |
| 1. Faculty of Agriculture 2. Faculty of Biotechnology and Biomolecular Sciences 3. Faculty of Computer Science and Information Technology 4. Faculty of Design and Architecture 5. Faculty of Engineering 6. Faculty of Medicine and Health Sciences 7. Faculty of Science 8. Institute of Bioscience | 1. Faculty of Agriculture and Food Sciences (UPMKB) 2. Faculty of Economics and Management 3. Faculty of Educational Studies 4. Faculty of Environmental Studies 5. Faculty of Food Science and Technology 6. Faculty of Forestry 7. Faculty of Human Ecology 8. Faculty of Modern Language and Communication 9. Faculty of Veterinary Medicine |

Note. Postgraduates from the selected faculties/institution represented individuals from the engineering and life sciences faculties/institution of Universiti Putra Malaysia, who share similar occupational lifestyle like any postgraduate students (young, dayshift working adults). They require high-level of morning alertness and concentration for their research works. They work mostly in WOPW with high reliance on architectural lighting throughout the day and need supportive workplace architectural lighting to perform their research experiments, observations, analyses and modeling (usually working with fine details using visual display units in sitting position).
