# Supplementary material for: Morning boost on individuals’ psychophysiological wellbeing indicators with supportive, dynamic lighting in windowless open-plan workplace in Malaysia
Source: PLoS One. 2018 Nov 29;13(11):e0207488. doi: 10.1371/journal.pone.0207488 (PMC6264480; doi:10.1371/journal.pone.0207488)
Supplement: S2 Table — Information on the scheduled experimental sessions (2 separate dates) and 12 IDs randomly assigned to each of the 4 EH light-setting. (DOCX) [file pone.0207488.s002.docx]

**S2 Table: Researcher’s reference list.** Information on the scheduled experimental sessions (2 separate dates) and 12 IDs randomly assigned to each of the 4 E_H_ light-setting.

| **E_H_ light-setting (lx)** | **Scheduled Dates** | **Allotted ID** |
| --- | --- | --- |
| 500-with-500: | Monday: | 001, 002, 009, 021, 023, 026, 027, 028, **034**, 037, 038, 045 |
| visit___1:___500_constant_500 | 01 Feb 2016 |  |
| visit___2:___500_constant_500 | 15 Feb 2016 |  |
| 500-with-250: | Tuesday: | 006, 010, 019, 020, 022, **024**, 029, 030, 031, 042, 047, 048 |
| visit 1: 500_decreased_to_250 | 02 Feb 2016 |  |
| visit 2: 250_increased_to_500 | 16 Feb 2016 |  |
| 500-with-750: | Wednesday: | 004, 005, 008, 011, 012, 013, 018, 033, 035, 039, 040, 041 |
| visit 1: 500_increased_to_750 | 03 Feb 2016 |  |
| visit 2: 750_decreased_to_500 | 17 Feb 2016 |  |
| 500-with-1000: | Thursday: | 003, 007, 014, 015, 016, 017, 025, 032, 036, **043**, 044, 046 |
| visit 1: 500_increased_to_1000 | 04 Feb 2016 |  |
| visit 2: 1000_decreased_to_500 | 18 Feb 2016 |  |

Note.

1. Before the briefing session, the researcher randomly allotted the 48 IDs (001 to 048) to the respective settings by drawing lots. The first ID drawn went to 500-with-500, second to 500-with-250, third to 500-with-750, fourth to 500-with-1000, and fifth back to 500-with-500, until all the IDs were drawn from an opaque container, resulting in an equal 12 IDs in each setting.
2. During the briefing session, IDs 024, 034 and 043 were not drawn by the participants as there were only 45 of them. This resulted in 12 participants in 500-with-750, and 11 participants each in 500-with-500, 500-with-250 and 500-with-1000.
