# Supplementary material for: Morning boost on individuals’ psychophysiological wellbeing indicators with supportive, dynamic lighting in windowless open-plan workplace in Malaysia
Source: PLoS One. 2018 Nov 29;13(11):e0207488. doi: 10.1371/journal.pone.0207488 (PMC6264480; doi:10.1371/journal.pone.0207488)
Supplement: S3 Table — (DOCX) [file pone.0207488.s003.docx]

**S3 Table. Summary of the light-setting’s immediate impact over time on the measured IPWI.**

| Indicators | Constant Lighting | | Dynamic Lighting | | | | | |
| --- | --- | --- | --- | --- | --- | --- | --- | --- |
|  | Constant (JKR standard) | | Increasing Oscillation | | | Decreasing Oscillation | | |
|  | visit 1: 500 _constant_ 500 lx  (control) | visit 2: 500 _constant_ 500 lx | visit 2: 250 _increased to_  500 lx | **visit 1: 500 _increased to_**  **750 lx** | **visit 1: 500 _increased to_**  **1000 lx** | visit 1: 500 _decreased to_  250 lx | visit 2: 750 _decreased to_  500 lx | visit 2: 1000 _decreased to_  500 lx |
| Urinary aMT6s | *** Y | *** Y | *** Y | *** Y | *** Y | *** Y | *** Y | *** Y |
| Alertness | * N | N | N | ^ Y | Y | * N | O | N |
| P_cog_ | ** Y | ** Y | ** Y | *** Y | *** Y | Y | * Y | N |
| PA | N | N | ** Y | * Y | Y | ** N | ^ N | * N |
| NA | ** Y | Y | * Y | * Y | * Y | * N | N | O |
| P_acuity_ | *** Y | ** Y | *** Y | *** Y | *** Y | ** Y | *** Y | *** Y |
| P_contrast_ | ** Y | ** Y | Y | Y | ** Y | Y | Y | Y |
| Visual Comfort | N | N | ** Y | Y | Y | *** N | * N | N |
| TOTAL Y | 5 | 5 | 7 | **8** | **8** | 4 | 4 | 3 |
| TOTAL N | 3 | 3 | 1 | 0 | 0 | 4 | 3 | 4 |
| TOTAL O | 0 | 0 | 0 | 0 | 0 | 0 | 1 | 1 |

Note. Information retrieved from Figs 5 - 9, panel 1.

Y = supportive change; N = unsupportive change; O = no change; *** p < 0.001, ** p < 0.01, * p < 0.05, ^ p < 0.06
