# Supplementary material for: Morning boost on individuals’ psychophysiological wellbeing indicators with supportive, dynamic lighting in windowless open-plan workplace in Malaysia
Source: PLoS One. 2018 Nov 29;13(11):e0207488. doi: 10.1371/journal.pone.0207488 (PMC6264480; doi:10.1371/journal.pone.0207488)
Supplement: S4 Table — (DOCX) [file pone.0207488.s004.docx]

**S4 Table. Summary of the light-setting’s immediate impact relative to control for the measured IPWI.**

| Indicators | Constant Lighting | | Dynamic Lighting | | | | | | |
| --- | --- | --- | --- | --- | --- | --- | --- | --- | --- |
|  | Constant (JKR standard) | | Increasing Oscillation | | | Decreasing Oscillation | | | |
|  | visit 1: 500 _constant_ 500 lx  (control) | visit 2: 500 _constant_ 500 lx | visit 2: 250 _increased to_  500 lx | **visit 1: 500 _increased to_**  **750 lx** | **visit 1: 500 _increased to_**  **1000 lx** | | **visit 1: 500 _decreased to_**  **250 lx** | **visit 2: 750 _decreased to_**  **500 lx** | **visit 2: 1000 _decreased to_**  **500 lx** |
| Urinary aMT6s | r | ^CI^ +ve | +ve | ^CI^ +ve | * +ve | | -ve | ^CI^ +ve | * +ve |
| Alertness | r | +ve | ^CI^ +ve | ** +ve | * +ve | | -ve | ^CI^ +ve | ^CI^ +ve |
| P_cog_ | r | -ve | -ve | ^CI^ +ve | ** +ve | | ^CI^ -ve | ^CI^ -ve | * -ve |
| PA | r | -ve | * +ve | * +ve | ^CI^ +ve | | * -ve | ^CI^ -ve | ^CI^ -ve |
| NA | r | ^CI^ -ve | ^CI^ -ve | ^CI^ -ve | -ve | | ** -ve | * -ve | * -ve |
| P_acuity_ | r | * -ve | ^CI^ -ve | +ve | ^CI^ -ve | | ^ -ve | ^CI^ -ve | ^CI^ -ve |
| P_contrast_ | r | +ve | ^CI^ -ve | * -ve | -ve | | * -ve | ^CI^ -ve | ^ -ve |
| Visual Comfort | r | -ve | * +ve | ^CI^ +ve | ^CI^ +ve | | *** -ve | ^CI^ -ve | ^CI^ -ve |
| TOTAL +ve  (nos. of inferred beneficial impacts) | 0 | 1 | 3 | **5** | **5** | | 0 | 2 | 2 |
| TOTAL -ve  (nos. of inferred non-beneficial impacts) | 0 | 2 | 3 | 2 | 1 | | **6** | **6** | **6** |

Note. Information retrieved from Figs 5 - 9, panel 2.

+ve = more supportive than control; -ve = less supportive than control; r = control; *** p < 0.001, ** p < 0.01, * p < 0.05, ^ p < 0.06

^CI^ +ve = wide CI that remained relatively large on the more supportive range, suggesting the confidence and possibilities of a beneficial impact.

^CI^ -ve = wide CI that remained relatively large on the less supportive range, suggesting the confidence and possibilities of a non-beneficial impact.
