# Supplementary figures and images for: Morning boost on individuals’ psychophysiological wellbeing indicators with supportive, dynamic lighting in windowless open-plan workplace in Malaysia
Source: PLoS One. 2018 Nov 29;13(11):e0207488. doi: 10.1371/journal.pone.0207488 (PMC6264480; doi:10.1371/journal.pone.0207488)

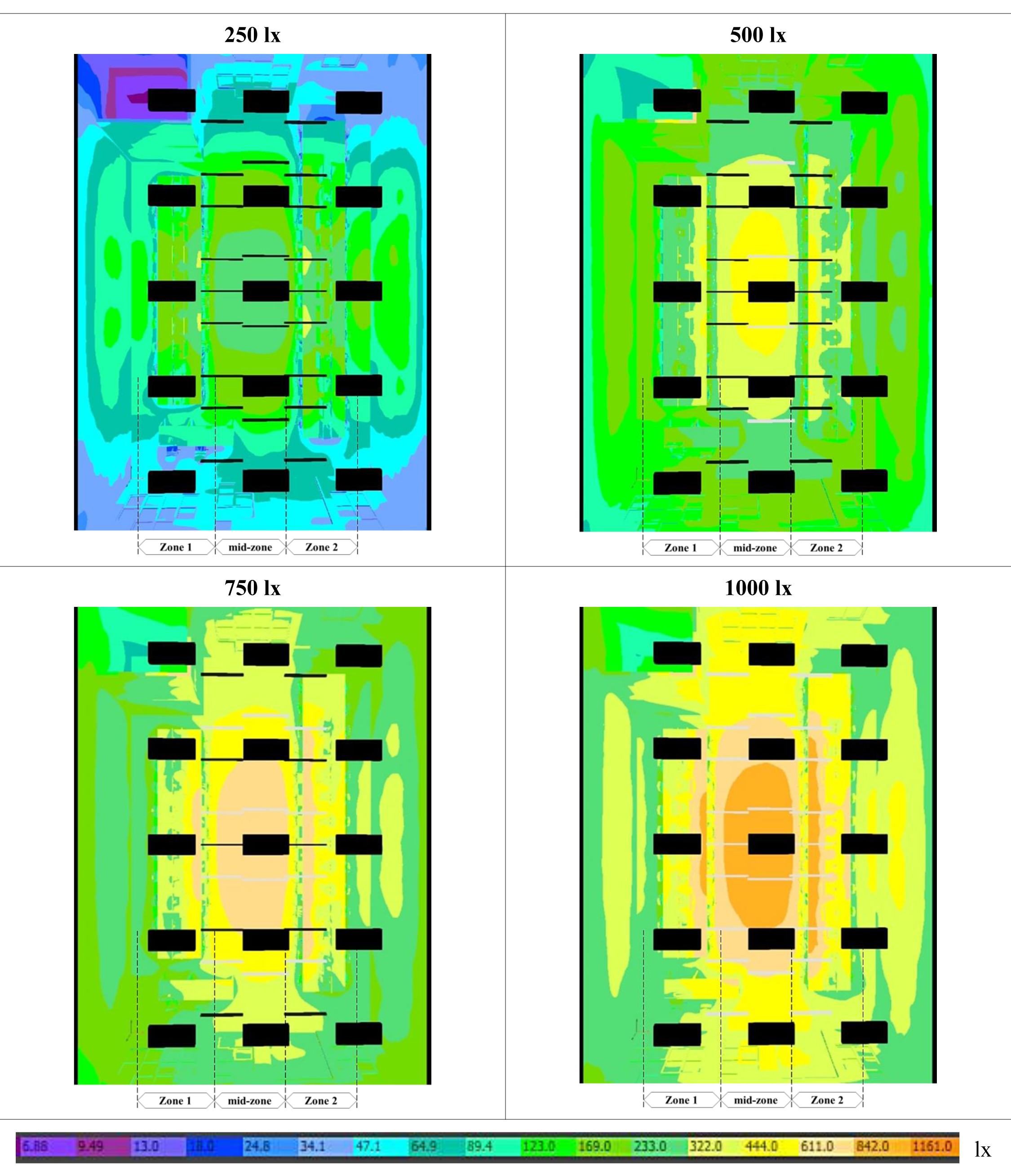

Supplement: S1 Fig — (TIF) [file pone.0207488.s005.tif]

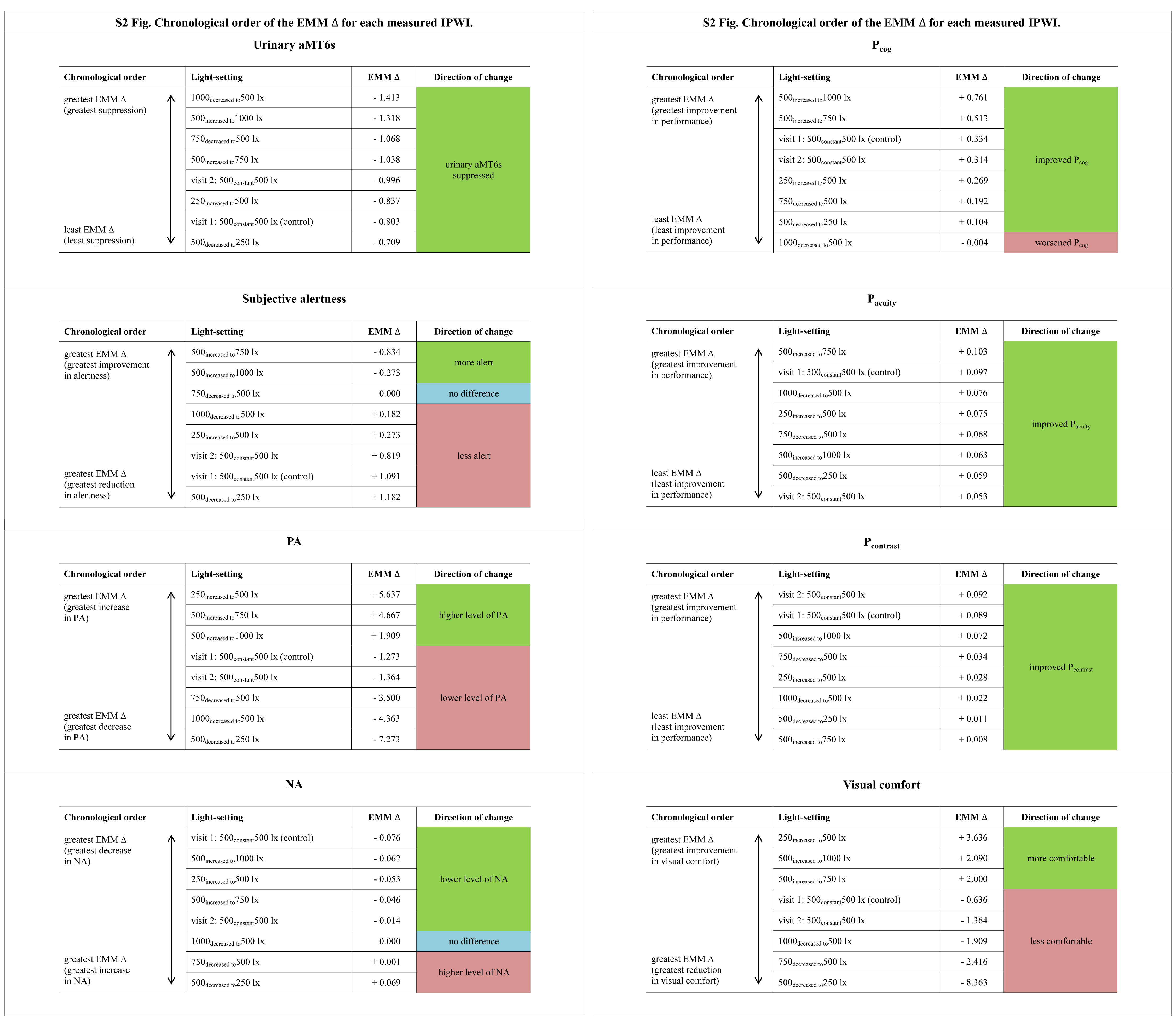

Supplement: S2 Fig — (TIF) [file pone.0207488.s006.tif]
