# Supplementary material for: Morning boost on individuals’ psychophysiological wellbeing indicators with supportive, dynamic lighting in windowless open-plan workplace in Malaysia
Source: PLoS One. 2018 Nov 29;13(11):e0207488. doi: 10.1371/journal.pone.0207488 (PMC6264480; doi:10.1371/journal.pone.0207488)
Supplement: S1 Appendix — (DOCX) [file pone.0207488.s007.docx]

**S1 Appendix. Specifications of the white LED lamp.**

| **Specification** | **Justification** |
| --- | --- |
| CCT: 6500 K  (Cool Daylight) | 1. Resembled a bright daylight ambient lighting condition, appropriate for the morning work period in Malaysia, and which is within the range of tropical daylight CCT (4000 to 20,000 K) [1]. 2. Recommended for tasks that demand alertness, high attention, and work output during working hours in Universiti Putra Malaysia [2]. 3. Visually preferred CCT for workplaces in Japan and sub-tropical regions [3,4]. |
| Color Rendering Index (CRI): 83 | 1. Malaysian Standard (MS 1525:2014) recommends CRI > 80 for workplace [5]. 2. Within the range of 80 to 90, which supports good color rendering properties [4]. |
| Spectral:  Polychromatic | 1. The blue wavelength peaked at 454 nm, which is within the 420 to 520 nm wavelength range suitable for melatonin suppression [6,7]. 2. The green-yellow wavelength humped at 558 nm suitable for photopic vision.   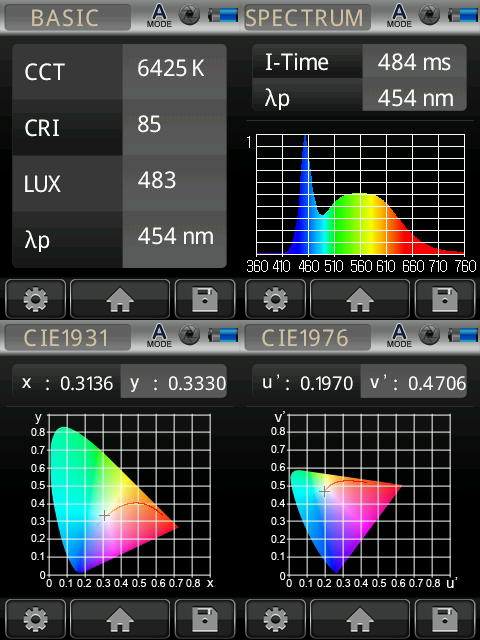  **S3 Fig. The spectral power distribution of Philips MASTER LEDtube.** |

# References in S1 Appendix

1. Sastri VDP, Das SR. Typical Spectral Distributions and Color for Tropical Daylight. J Opt Soc Am. 1968;58: 391. doi:10.1364/JOSA.58.000391

2. Shamsul B, Sia CC, Ng YG, Karmegan K. Effects of Light’s Colour Temperatures on Visual Comfort Level, Task Performances, and Alertness among Students. Am J Public Heal Res. 2013;1: 159–165. doi:10.12691/ajphr-1-7-3

3. Akashi Y, Boyce PR. A field study of illuminance reduction. Energy Build. 2006;38: 588–599. doi:10.1016/j.enbuild.2005.09.005

4. Basics of light and lighting [Internet]. Koninklijke Philips Electronics N.V. 2008. Available: www.philips.com

5. Malaysian Standard: Energy Efficiency and Use of Renewable Energy for Non-Residential Buildings - Code of Practice (MS 1525:2014). Department of Standards Malaysia (STANDARDS MALAYSIA) Malaysia; 2014 pp. 1–46.

6. Rea MS, Bullough JD, Figueiro MG. Phototransduction for human melatonin suppression. J Pineal Res. 2002;32: 209–13. doi:10.1034/j.1600-079X.2002.01881.x

7. van Bommel WJM. Non-visual biological effect of lighting and the practical meaning for lighting for work. Appl Ergon. 2006;37: 461–466. doi:10.1016/j.apergo.2006.04.009
