# Supplementary material for: Morning boost on individuals’ psychophysiological wellbeing indicators with supportive, dynamic lighting in windowless open-plan workplace in Malaysia
Source: PLoS One. 2018 Nov 29;13(11):e0207488. doi: 10.1371/journal.pone.0207488 (PMC6264480; doi:10.1371/journal.pone.0207488)
Supplement: S2 Appendix — (DOCX) [file pone.0207488.s008.docx]

**S2 Appendix. Overview of the visual comfort assessment and its scoring.**

This modified questionnaire consisted of 13 items, designed to understand the participant’s perception (*P*), satisfaction/dissatisfaction and experiences (*S&E*) about the lighting conditions in the laboratory. Three of the items were related to *P* inquiries, and their responses were multiple choices. The remaining 10 items were *S&E* inquiries, with a symmetrical 2-stage scale response (yes/no) without any neutral choices; which resembled the dichotomous response (agree/disagree) found in the original Office Lighting Survey [1]. This study posited the dichotomous response was adequate to gain preliminary empirical data [2,3], and help minimize possible misinterpretations associated with neutral choices [4]. Scores were assigned to every response based on the style of inquiry.

| **Type** | **Item** | **Question / Statement** | **Inquiry Style** | **Response** | **Scoring** |
| --- | --- | --- | --- | --- | --- |
| *P* | 1. | Rate the level of brightness. | n.r. | Too dim (too dark) | -2 |
|  |  |  |  | Dim (dark) | -1 |
|  |  |  |  | Just right | 0 |
|  |  |  |  | Bright | +1 |
|  |  |  |  | Too bright | +2 |
|  | 11. | How would you describe the lighting condition at this workstation, compared to your usual workstation in UPM campus? | n.r. | Worse | -1 |
|  |  |  |  | No Difference | 0 |
|  |  |  |  | Better | +1 |
|  | 12. | As part of a 4-hour work day in the morning (8am to 12pm), how long do you think you can work under this lighting condition? | n.r. | Less than 1 hour | 0 |
|  |  |  |  | 1 to 2 hours | +1 |
|  |  |  |  | 2 to 3 hours | +2 |
|  |  |  |  | 3 to 4 hours | +3 |
| *S&E* | 2. | Are you able to tolerate the glare? | +ve | Yes | +1 |
|  | 3. | Are you comfortable with this lighting condition? |  |  |  |
|  | 4. | Can you concentrate easily in this brightness? |  |  |  |
|  | 6. | Can you see clearly while doing this computer-based work? |  | No | -1 |
|  | 13. | Is brightness of light in your workplace important for your wellbeing? |  |  |  |
|  | 5. | Is the reflection delaying your work output? | -ve | Yes | -1 |
|  | 7. | I experience headache working under this lighting condition. |  |  |  |
|  | 8. | I experience eyestrain when working under this lighting condition. |  |  |  |
|  |  |  |  | No | +1 |
|  | 9. | I experience affected vision when working under this lighting condition. |  |  |  |
|  | 10. | I experience giddiness working under this lighting condition. |  |  |  |

# References in S2 Appendix

1. Eklund NH, Boyce PR. The Development of a Reliable, Valid, and Simple Office Lighting Survey. J Illum Eng Soc. 1996;25: 25–40. doi:10.1080/00994480.1996.10748145

2. Aday LA, Cornelius LJ. Designing and Conducting Health Surveys: A Comprehensive Guide. 3rd ed. San Francisco: Jossey-Bass; 2006.

3. Akashi Y, Boyce PR. A field study of illuminance reduction. Energy Build. 2006;38: 588–599. doi:10.1016/j.enbuild.2005.09.005

4. Linhart F, Scartezzini J-L. Evening office lighting - visual comfort vs. energy efficiency vs. performance? Build Environ. 2011;46: 981–989. doi:10.1016/j.buildenv.2010.10.002
